# How outcome prediction could affect patient decision making in knee replacements: a qualitative study

**DOI:** 10.1186/s12891-016-1165-x

**Published:** 2016-07-22

**Authors:** Timothy Barlow, Patricia Scott, Damian Griffin, Alba Realpe

**Affiliations:** CSB, University of Warwick, UHCW, Clifford Bridge Road, Coventry, CV2 2DX UK

**Keywords:** Patients’ decision making, Qualitative, Knee replacement, Outcome prediction

## Abstract

**Background:**

There is approximately a 17 % dissatisfaction rate with knee replacements. Calls for tools that can pre-operatively identify patients at risk of being dissatisfied have been widespread. However, it is not known how to present such information to patients, how it would affect their decision making process, and at what part of the pathway such a tool should be used.

**Methods:**

Using focus groups involving 12 participants and in-depth interviews with 10 participants, we examined how individual predictions of outcome could affect patients’ decision making by providing fictitious predictions to patients at different stages of treatment. A thematic analysis was used to analyse the data.

**Results:**

Our results demonstrate several interesting findings. Firstly, patients who have received information from friends and family are unwilling to adjust their expectation of outcome down (i.e. to a worse outcome), but highly willing to adjust it up (to a better outcome). This is an example of the optimism bias, and suggests that the effect on expectation of a poor outcome prediction would be blunted. Secondly, patients generally wanted a “bottom line” outcome, rather than lots of detail. Thirdly, patients who were earlier in their treatment for osteoarthritis were more likely to find the information useful, and it was more likely to affect their decision, than patients later in their treatment pathway.

**Conclusion:**

This research suggest that an outcome prediction tool would have most effect targeted towards people at the start of their treatment pathway, with a “bottom line” prediction of outcome. However, any effect on expectation and decision making of a poor outcome prediction is likely to be blunted by the optimism bias. These findings merit replication in a larger sample size.

**Electronic supplementary material:**

The online version of this article (doi:10.1186/s12891-016-1165-x) contains supplementary material, which is available to authorized users.

## Background

Primary osteoarthritis (OA) of the knee causes loss of function, pain, and deterioration in quality of life. This leads to difficulty in working and performing activities of daily living, stress, and depression [1]. Knee OA affects 10 % of the UK population over 55 years [2]. The number of people with this problem is increasing as the population ages. Total Knee Replacement (TKR) has been shown to have a reliably beneficial effect [3], and around 90,000 primary TKRs were performed in England and Wales in 2014, with over 95 % for OA [4]. Although TKRs are expensive, they are one of the most cost effective interventions for any illness or disease [3].

However, questions have been raised about the benefits of TKRs: some studies report up to 17 % of patients are dissatisfied with the outcome of knee replacement surgery [5–7]. The situation has recently been highlighted by the Health Secretary, and resulted in some commissioning bodies reducing access to this treatment [8].

In response to this, the identification of patients at risk of poorer outcomes has been assigned as a research priority by various bodies including the British Orthopaedic Association, Arthritis Research U.K., and the National Institute for Health and Care Excellence (NICE) [9, 10].

Many previous studies have attempted to identify predictors of outcome, examining various factors including surgical factors and patient factors [7, 11–16]. To date, none have been successful in developing a tool that can usefully predict outcome; however, there are currently several investigations into the development of such a tool, and it appears that psychological factors account for a large amount of the variability in outcome [7, 17–19].

An outcome prediction tool would have the potential to provide patients with an individualised prediction of outcome. This has broad implications, including the management of pre-operative expectation (potentially improving post operative satisfaction based on reducing any disparity between expectations and outcome), helping improve decision making about progressing to a knee replacement, and facilitating investigation into interventions to improve outcome in patients who have worse predictions (likely psychological interventions).

Alongside this quantitative work research into understanding what factors are important in patients decision making has gained momentum. Multiple studies across multiple countries have demonstrated a remarkable consistency in important factors that influence patients’ decision making [20]. A key concept, and relevant to outcome prediction, is that of the Deliberation/Determination model proposed by Elwyn [21]. This splits decision making into a Deliberation phase, and a Determination phase. Additional studies have demonstrated a “decision making threshold” – a moving target of the point at which a patient changes from deliberating the decision to making the decision, usually the point at which coping with the status quo is no longer acceptable [22, 23].

However, what is unclear, and has never been studied before, is how individual prediction of outcome would affect patients’ decision making. The distinction between this sort of tool and current Patient Decision Aids (PDAs) that are available is important: PDAs act as a means of describing current knowledge about a condition and treatment options to help patients make decisions. PDAs provide no extra information than that which is already available, and would likely be discussed between a surgeon and a patient [24]. Critically, PDAs do not give a prediction of outcome for individual patients [24].

In anticipation that we can one day predict poor outcomes, we wanted to explore the potential value of a prediction tool for patients: specifically, we were interested in the content and presentation of information that patients would want, patients’ views on how a tool would affect their expectations and decision making, patients’ views on if a tool would be perceived to reflect an “unhealthy psychology” (given the large effect of psychological factors on outcome), and the acceptability of offering alternative treatments that could address modifiable (and likely psychological factors) before having a knee replacement.

## Methods

This study occurred in parallel with a separate qualitative study, using the same patient group. Two stages were performed: focus groups to generate a range of patient views and in-depth interviews to explore those views in depth [25]. Focus groups took place with patients who had already had a knee replacement, and interviews took place with patients who were either waiting for a knee replacement, or considering having one. Examining these three points in the patient pathway allowed us to investigate the two stages of the decision making process proposed by Elwyn, Deliberation and Determination [21]. No sample size was specified before the sample began, but previous reports examining decision making and using similar methodology reached saturation at around 10–20 patients [20].

### Interview and focus group conduct

TB, an experienced qualitative researcher and orthopaedic surgeon in training, conducted all focus groups and interviews. Focus groups were facilitated by AA or AR, both experienced qualitative researchers. PS, a member of the public with little qualitative experience, was present for focus groups and four interviews.

Focus groups and interviews took place at UHCW, the patients home, and by telephone based on patient preference.

All patients were provided with a paper copy of a fictitious report. Patients were aware the report was fictitious. There were multiple versions of this report, which evolved based on patient feedback. Key aspects included a summary of predicted pain post operatively and a summary of predicted function post operatively. Text and graphics were used, with comprehension and preferences noted. Participants were made aware of how such a report may be generated (especially the involvement of psychological factors). An example of a report can be found in the Additional file 1.

### Sampling

Purposive sampling to ensure a range of ages and genders was conducted. Socioeconomic status (Index of Multiple Deprivation 2007) and ethnicity were monitored; however, they did not contribute to purposive sampling [26].

### Focus groups

Patients who had a previous total knee replacement for knee osteoarthritis at University Hospitals of Coventry and Warwickshire (UHCW) on or before 30th April 2013 were identified through medical coding. No patient was excluded on the basis of outcome or complications. 100 invitation letters for focus groups were sent in October 2013 or February 2014 using an “opt in” approach. Focus groups took place in December 2013 and March 2014. A schedule is available in the Additional file 2.

### In-depth interviews

TB, AA and PS analysed the focus groups and produced the interview schedule (see “Analysis” below). The schedule (see Additional file 3) was almost identical to the focus group schedule.

During the interviews two different points on the patient pathway was targeted. The first used an “opt-in” method with purposive sampling to identify patients who had either had a knee arthroscopy, or were waiting for one. Patients were identified through medical coding and sent invitation letters from October 2014 to December 2014. Only patients with a diagnosis of osteoarthritis were included (this represents over 95 % of the patients receiving knee replacements, and is where the majority of effort is being directed in developing an outcome prediction tool) [4, 7, 18]. This population represented patients who had osteoarthritis of the knee and were being seen in secondary care, but had not decided to have a knee replacement (Deliberation phase).

The second targeted patients who were participating in a multi-centre cohort study designed to develop an outcome prediction tool [18]. These patients had knee osteoarthritis, were over 50 years old (as almost all candidates for knee replacement are), and were on the waiting list for a knee replacement. This group of patients were approached from the 4th to the 11th of July 2014 and invited to participate, and represent patients in the Determination phase of decision making.

### Conduct of focus groups/interviews

The usefulness of information an outcome prediction tool may be able to provide was tested with all participants via the use of a fictitious report containing information that an outcome prediction tool may contain (please see Additional file 3). This report evolved based on feedback. How the tool could affect the decision making process overall, and its effect on factors involved in decision making, were explored. Specific questions, based around topics the research team thought would be important, were asked, but participants were encouraged to provide their own views and options. Pre-defined areas thought critical to cover included perceived benefits of the tool, perceived sensitivities, preference of delivery of tool, its effect on decision making, and patients’ acceptability of being offered alternative treatment on the basis of their personal prediction. Patients’ view of alternative treatment was considered relevant as psychological factors appear to account for a large amount of the variability in outcome. Therefore alternative treatment is likely to surround psychological therapies, and may be associated with stigma or relatively fixed views [11].

### Analysis

Thematic analysis was used to analyse the data: this term has been used in many different situations to describe different approaches to qualitative data analysis [27]. For the purposes of this research project, with the authors coming from a predominantly realist perspective (the idea we interact with a real world and our theories refer to that world), this involved an inductive (bottom up) thematic analysis with a predominantly semantic development of themes [25]. However, interpretation of all themes and subthemes was undertaken in an attempt to conjecture the wider meanings of the patterns emerging from the data [27].

Analysis was performed by TB, AA and PS. PS has no specific training in the analysis of qualitative data, but her role in providing a member of the public’s opinion was invaluable.

All focus groups and interviews were transcribed and data were organized with the help of computer software [28], with the exception of one participant of an interview who declined to have the conversation recorded; therefore the interviewer’s notes were used for analysis.

Each member of the team contributed to the development of a coding framework. Potential themes identified by each researcher were discussed and agreed upon by regular meeting of the research team, including our public representative. The process of searching for themes, reviewing themes, and defining and naming themes was conducted in line with recommendations of Braun and Clarke [27]. Analysis and data collection continues simultaneously, particularly relevant to the analysis for the focus groups, which informed the development of the indepth interview guide. This iterative approach to data collection and analysis allowed full exploration of emerging themes. Data collection stopped when no new themes were emerging from the data collected. When necessary, transcripts that had already been coded were revisited when a modification of the coding framework and themes took place.

AR cross-referenced 10 % of the interview data (randomly selected) to test the validity and reliability of the coding data. Reliability statistics (percentage agreement and Cohen’s Kappa) were calculated by software available online [29]. Instances of one coder using multiple references when the other coder had included one larger reference were resolved by using the main coding topic for each coder and including it as one variable.

Various methods were used to improve trustworthiness. Credibility has been addressed by triangulation of decision making stage and member checking (of both transcripts and concepts that had been derived from them) [30]. Additionally, the research team felt that the participants were very open, especially within the focus groups. Although focus group setting may be considered harder to gain an inclusive and open dialogue due to group dynamics [25], we found frank and open discourse with an abundance of personal and sensitive information disclosed for the aid of the conversation. This was undoubtedly helped by the involvement of PS, a member of the public and advisor on the study. We have provided a thick description of the setting, situation, times, and people to address issues of transferability; however, a caveat exists in that all participants in our study were engaged with secondary care. Dependability is closely tied to credibility, and the use of “overlapping methods” of focus groups and interviews combined with detailed description of the study process has helped to address this [30].

## Results

### Patient numbers and demographics

**Fig. 1:**
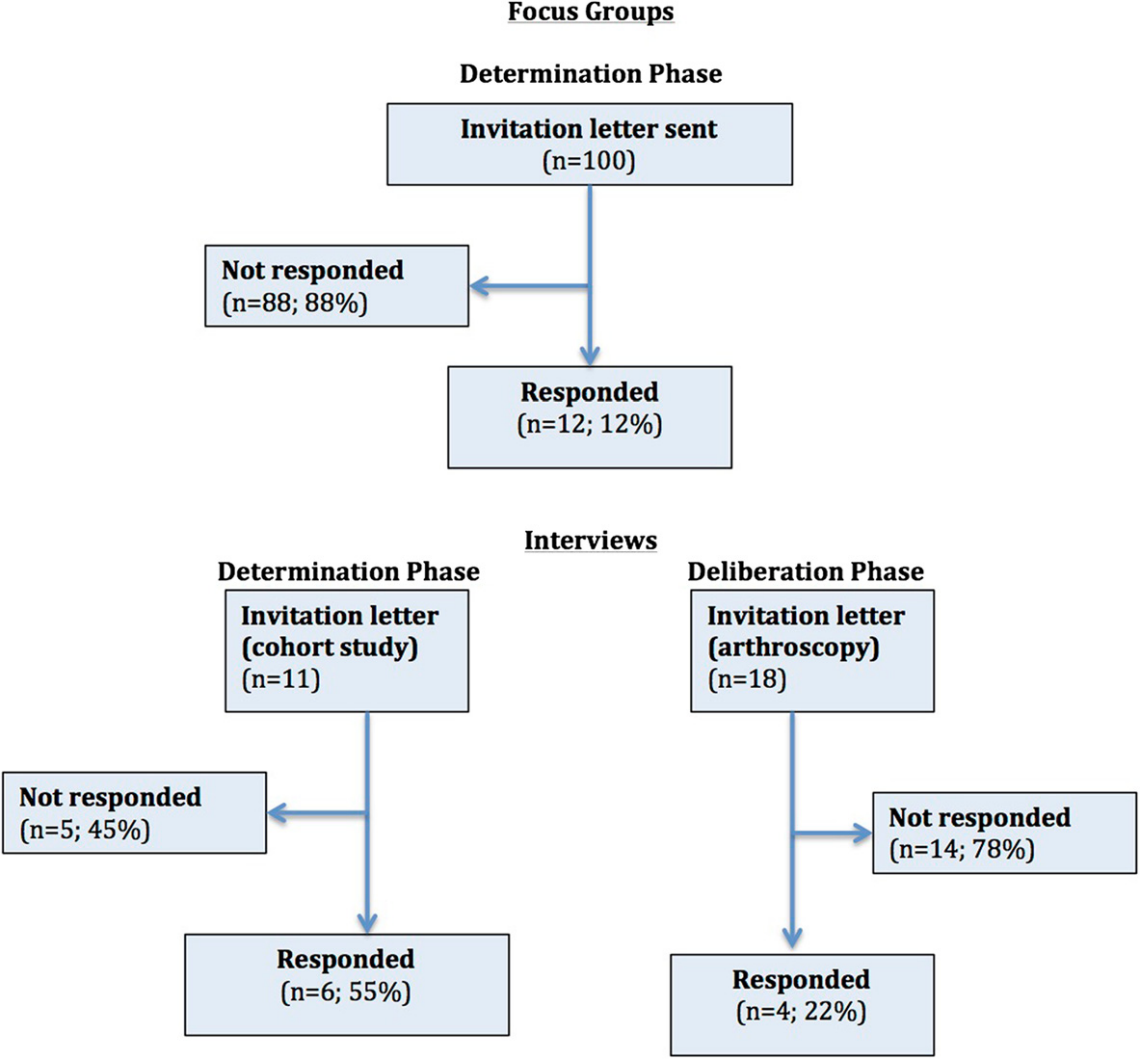
Flow diagram of included patients

Six patients took part in two focus groups (12 patients total). This represents a 12 % response rate to the “opt in” letters. Eleven patients taking part in a cohort study developing an outcome prediction tool for patients considering a knee replacement were approached [18], with six agreeing to take part (Determination phase). Eighteen patients either waiting for or having received a knee arthroscopy were approached for the Deliberation phase interviews, with four taking part (20 %). A flow diagram of invited and included patients can be found in Fig. 1.

Tables 1 and 2 demonstrate the demographic breakdown of patients involved focus groups and interviews respectively. Only 5 % of patients identified for interviews were of Asian origin, which does not reflect the population that UHCW serves (12–13 % Asian origin) [31].

**Table 1:** Demographics of participants in focus groups

Patient	Gender	Age	Ethnicity	Sociodemographic class^a^ (decile)
1	M	72	White British	17882 (5)
2	F	76	White British	20924 (6)
3	M	71	White British	22203 (6)
4	F	68	Indian	21358 (6)
5	F	71	White British	18732 (5)
6	F	67	White British	26766 (8)
7	M	76	White British	12697 (3)
8	M	72	White British	13458 (4)
9	F	57	White British	16472 (5)
10	F	77	White British	182 (1)
11	M	72	White British	22835 (7)
12	F	82	White British	18702 (5)

^a^Using the Index of Multiple Deprivation 2010 ranks for Lower lay Super Output Areas (LSOA) (1 = most deprived, 32,482 = least deprived). Decile – data ranked from 1 (highest level of deprivation) to 10 9lowest level of deprivation) by dividing into 10 equal groups

**Table 2:** Demographics of participants in interviews

Patient	Stage of decision making	Gender	Age	Ethnicity	Sociodemographic class^a^ (by decile)
1	Post (waiting list)	F	68	White British	31755 (9)
2	Post (waiting list)	M	64	White British	18479 (5)
3	Post (waiting list)	M	68	White British	32096 (9)
4	Post (waiting list)	M	78	White British	30195 (9)
5	Post (waiting list)	F	52	White Other	26469 (8)
6	Post (waiting list)	M	63	White British	24905 (7)
7	Pre	M	73	White British	22006 (7)
8	Pre	F	70	White British	22552 (7)
9	Pre	M	51	Asian	
10	Pre	M	53	White British	

^a^Using the Index of Multiple Deprivation 2010 ranks for Lower lay Super Output Areas (LSOA) (1 = most deprived, 32,482 = least deprived)

### Reliability

Percentage agreement in the 10 % of the interview data checked was 77 %, with Cohen’s Kappa 0.72. This represents a “satisfactory” level of agreement, using both a liberal and conservative measure of reliability [29].

### Thematic analysis

We identified six major themes within our study. Five of these themes were those identified as potentially important before the study began (perceived benefits of the tool, perceived sensitivities, preference of delivery of tool, its effect on decision making, and patients acceptability of being offered alternative treatment on the basis of their personal prediction) and one was additional (optimism bias).

#### Benefits of outcome prediction

Participants were universally positive about the principle behind the tool, feeling that having such information would be helpful:

“If they said that you were going to be pain-free but your functionality wasn’t going to be as good, you may not be able to bend it and you would have to walk with a stick. Or if they said the opposite, you’re still going to have some pain but your functionality is going to be a lot better then you’ve got some information to make a decision.” (Focus Group 2; Determination phase)

There was also the belief that having information in a written format that could be taken away was a worthwhile aim, especially for people who are socially isolated and may not have the contacts with friends or family to discuss outcome:

“It would certainly fill that gap. I mean the fact that we’ve got lots of family here and down in the south lots of other Africans, it’s not really a factor but just thinking of that sort of, there are lots of other people that have come to live here from other countries that don’t have a support group , I think that would be really beneficial.” (Interview 1; Determination phase)

The type of information the tool conveyed also had positive effects. This was true for predictions that were on the whole positive, where respondents felt it gave them confidence to proceed:

“Yes I, I, yes. I’d feel great….Yes give me confidence.” (Interview 5; Determination phase)

Interestingly, and quite unexpected, were the positive aspects of providing a report that was predominantly negative. One patient felt that it would have been easier to cope with a poor result, as she would have blamed herself for it less:

“And he kept saying, “I can’t believe how bad you are.” And I said, “Neither can I.” …;” **I probably wouldn’t have been quite as hard on myself,** because I kept thinking, “Well, what’s gone wrong?”” (Focus Group 2)

Sources of information have been identified in previous studies as a key aspect of patients decision making [20] and a further effect of the report was that people felt it would likely affect the sources of information that they went to, but they would not use it as a sole source:

“If I had got that before…because I’d done all that before I came to the knee clinic, and for my pre-op, so I still would have asked my friend, but I think I wouldn’t have bothered looking on Google at the different things.” (Interview 1; Determination phase)

#### Sensitivities related to tool

One focus of enquiry that was identified before the study began by the research team was that of a poor prediction of outcome being related by the patients to a diagnosis of an “unhealthy psychology”. This was based on the fact that psychological factors are the biggest predictor of outcome that are known to date (although the majority of variability is still unaccounted for) [11]. All participants were asked to give their thoughts on this. No participant displayed any concerns regarding a poor prediction being associated with an “unhealthy psychology”:

“No I don’t think it would do anything with mental health, no.” (Interview 2; Determination phase)

This quote is particularly relevant, as it was from a participant in a research study developing an outcome prediction tool by examining predominantly psychological factors. Having undergone a barrage of psychometric tests, the participant did not make a link between a poor outcome and psychological wellbeing.

The second focus of enquiry that was identified before the study began was that of patients being aware that a prediction was not a guarantee of outcome. All study participants appreciated this:

“No, it’s not. The way you’ve explained it in there as well, then you’re not guaranteeing it, it should be. You’re not guaranteeing it.” (Interview 8; Deliberation phase)

However, six participants expressed concern that not all people would see it the way they did:

“you’re going to get some comeback .. he’s gone the opposite way round, and you’ve predicted this, that’s where you’re going to get your comeback. Because they’re not going to be happy with what you predicted.” (Interview 4; Determination phase)

A further concern of patients was the use of this information to rationalize or prioritise patients for theatre. Some people thought that this was a reasonable course of action, assuming that “the 20 %” who have a poorer outcome should be prioritised:

“But presumably these lists are prioritised using a whole variety of criteria and this is just adding to this criteria, surely. They would still have to be prioritised; the most urgent get done first.” (Focus Group 2; Determination phase)

However, others were very concerned that this sort of information should not be used for either rationalization or prioritization, and should only be used to provide information to patients:

“R: Well it’s a process for the patient, it’s not a process for the surgeon…”

“I: It would predict what your eventual outcome would be. And should the surgeon use that when rationalising whether you should have a knee replacement or not?

R: The answer in my opinion is ‘no’.” (Focus Group 2; Determination phase)

However, there was concern that a poor report was a surprise and that such information could “frighten people away”.

“it isn’t what I would have understood heard or expected.” (Interview 3; Determination phase)

“don’t know about that, I don’t know, I think you have got to be very careful that you don’t frighten people away from what needs to be done.” (Interview 6; Determination phase)

#### Preferred delivery of tool

There are differences in the amount of information that different patients want in a wide range of medical situations [32–34]. When it came to the outcome prediction tool there was a general preference for a “bottom line” approach and visual displays:

“That would have been brilliant … I like the picture that says in all probability you may expect an improvement of X per cent, I think that’s the way I would like to receive the information.” (Focus Group 1; Determination phase)

“I think the fact that it’s a visual aid is helpful. I think again, rather than simply having script, to have a visual aid is almost essential. I know I can do that. You can see it physically.” (Focus Group 2; Determination phase)

There was a feeling that people would need someone to go through this information with them in a face to face context:

“My instinct says it’s better to have somebody to go through it with you. I think just receiving it … I found not the easiest thing I’ve done in the last seven days … There are some people who can’t speak English or understand, they have to be explained and all that.” (Focus Group 1; Determination phase)

Trust in the output from the tool was also seen as essential:

“I think you’d need to know where the figures are coming from … one knows that these are just the opinions or whether they are from a clinical analysis or something, you know” (Interview 6; Determination phase)

Therefore a general preference for graphical displays with a bottom line was present, along with the opportunity to discuss it with a medical professional.

#### The tool’s effect on decision making

The tool’s effect on decision making was tested on three different groups of people, each at a different stage of the decision making process: those in the Deliberation phase; those just after making a decision, but still waiting for the operation (Determination phase); and those who had already had the operation (Determination phase). The effect of the tool was examined in each group

##### Patients who had already had an operation

In this group patients were asked how the tool would have affected their Deliberation process, and therefore the difficulties inherent with any retrospective inquiry were present. However, all patients thought that the information would have had the ability to change their expectations:

“I: How would it have affected your thinking process?

R: No, I think weighing up the pros and cons and if you’re in a lot of pain I think you just go for it.

R: You’d lower your expectations wouldn’t you? (Another member of focus group asking question)” (Focus Group 2; Determination phase)

It was challenging to get this group of patients to distinguish between altering their decisions, and altering their expectations. Interestingly around half of patients felt strongly it would have had no effect on their expectations if the predicted outcome was poor, but would have improved confidence if the predicted outcome was good. This will be discussed in more detail under “optimism bias”.

There was a predominant feeling that the tool would not have affected the decision:

“No, likewise, the same as well, it wouldn’t have made any difference because I was in pain and it needed to be done.” (Focus Group 1; Determination phase)

However, only four people thought that it would have helped the decision making process:

“I: do you think [an outcome prediction tool] would have helped?

R: Possibly; it would have been a lot easier.” (Focus Group 2; Determination phase)

##### Patients who were on the waiting list for a knee replacement

This group of patients universally would have changed their expectations; however, again we saw a division on if it would have affected the Deliberation phase, with around half of patients stating it would have:

“I think I certainly would have thought instead of that initial response when he told me I needed to have it, I might have said I need to think about it a little bit first,” (Interview 1; Determination phase)

And others stating it would not; however, this was less strongly held than the post-operative group, often with a qualifier:

“it would have been nice to know but in, in my situation no it wouldn’t have [altered my decision].” (interview 5; Determination phase)

##### Contemplating knee replacement

In this group all patients felt that the information in the outcome prediction tool would have affected their expectation and their Deliberation. This was a strongly held belief:

“This sort of information are enough to change anybody’s mind” (Interview 10: Deliberation phase)

“Yes, of course it affects expectations” (Interview 10; Deliberation phase)

Overall there was a stark difference in how the tool affects decision making. This result is not unexpected and could be due to two effects, which is explored in the discussion.

When patients were asked directly when they thought this information should be given there was a complete range of responses:

“I think I would like the GP to action this and get it through.” (Focus Group 1; Determination phase)

“Pre-op assessment.” (Focus Group 1; Determination phase)

“I think the consultant or the consultant’s team is or are the best people.” (Focus Group 2; Determination phase)

#### Acceptability of alternative treatment

Alternative treatment (e.g. Cognitive Behavioral Therapy (CBT)) has the potential to improve the outcome from knee replacements. CBT can alter psychological processes, such as coping strategies. These factors could affect outcome in knee replacement and by modifying them before knee replacements, outcome could be improved. This (highly theoretical) option was posed to patients by asking if they would be willing to delay their operation to undergo CBT if it would be likely to improve their outcome. A remarkable division between pre and post operative patients resulted, with post operative patients universally disagreeing with any delay:

“I don’t want to suffer another three months. Go for it, I think.” (Focus Group 1; Determination phase)

And pre-operative patients having mixed views:

“Well I suppose I would then if you felt that I … that’s what I needed…Yes. I mean you have to listen to the medical staff, they do … you know to some degree know a bit better than you do.” (Interview 3; Determination phase)

“I wouldn’t have wanted it to be delayed.” (Interview 1; Determination phase)

Participants were generally not particularly receptive to psychological therapies, which is consistent with the participants’ view that a poor prediction of outcome was not associated with an “unhealthy psychology”.

#### Optimism bias

Sharot defines the optimism bias as the propensity to:

“*Overestimate the likelihood of positive events, and underestimate the likelihood of negative events. For example, we underrate our chances of getting divorced, being in a car accident, or suffering from cancer. We also expect to live longer than objective measures would warrant, overestimate our success in the job market, and believe that our children will be especially talented. This phenomenon is known as the optimism bias, and it is one of the most consistent, prevalent, and robust biases documented in psychology and behavioural economics*.” [35]

We found that patients who have already had a knee replacement were unwilling to move their expectations down with a poor prediction of outcome, but willing to have a good prediction inspire confidence. The finding of willingness to accept the prediction when it was good, combined with an unwillingness when it was bad, was present across all three groups, suggesting that the optimism bias is inherent in this decision and is consistent with literature examining preference based decisions [35].

“No because I know within myself you know I’m a pretty healthy guy and I’d like to think I’d got a lot more than.” (Interview 5; Determination phase)

“And if your friend had said it’s fantastic, but you’d got this bad report, who would you have trusted?

R: I would have trusted her, but I would also everyone’s different and our bodies are all different so I think I would have probably gone in the middle and my expectations would have been a bit of both.” (Interview 1; Determination phase)

And with a positive outcome:

“R: Well I trust him, I’d believe him.

I: You’d believe it?

R: I’d believe him because I wanted to..” (Interview 8: Determination phase)

This has implications for the implementation of such a tool, which will be explored in the discussion.

## Discussion

This study has suggested that the effects of any future outcome prediction tool on patients decision making will depend on their stage of decision making (Deliberation or Determination), and that the effects of a poor outcome prediction may be blunted by the optimism bias.

Overall there was a stark difference in how the tool affects decision making at different stages of the decision making process. This result is not unexpected and could be due to two effects. Firstly, the Deliberation phase of decision making is stressful, and there is a relief once a decision is made [36]. Therefore, any new information that comes to light once the decision is made is less likely to change that persons mind. The stress from the Deliberation phase appears to act as a barrier to revisiting an already made decision. This is critical when considering at what point in the pathway of care an outcome prediction tool should be used. Secondly, there could be a hindsight bias, where people are offering explanations that are coherent with their behaviours and outcomes (also described as part of the wider cognitive dissonance theory).

The optimism bias has implications on the effect an outcome prediction tool would have on the high dissatisfaction rate seen with knee replacements. The reason an outcome prediction tool has been allocated as a research priority by NICE is the high dissatisfaction rate. It is this group that the tool would be most useful as it would alter expectations in line with predicted outcome and, potentially, result in improved satisfaction. However, what we have found here is that although patients would alter their expectations, they would not alter them all the way towards a “bad” prediction. This is likely to dampen the effect on expectations and decision making an outcome prediction tool could have for patients. Additionally, the link between expectations and satisfaction in knee replacement, although logically consistent, has been called into question in a recent systematic review [37]. However, the authors of this paper warn that differences in measuring constructs such as expectations and satisfaction make conclusions prone to error.

The stage of decision making and the optimism bias has broad implications for any future outcome prediction tool. The effect of the tool on managing expectations and patient decision making will alter depending on the point in the pathway it is used, and the effect of the tool may not be as great as the orthopaedic community at large hope. However, it is clear that patients welcome such information, and that it would appear to still have an effect on expectation and decision making, especially if targeted early in the patient pathway.

The strengths of this study include: its focus on one area of decision making, namely preference based decision making in total knee replacement; the inclusion of people at various stages of the decision making process; the broad based research team, including a member of the public who was involved at all stages; the inclusion of a range of ages, genders, and ethnicities within the study; the comprehensive analysis; and thoughtful efforts to demonstrate the trustworthiness through techniques to improve the credibility, transferability, dependability, and conformability of the study.

Due to our sample size, we expected the focus groups to include both patients who perceived themselves as having good outcomes and bad outcomes. This allowed a broader range of perspectives to be included; however, with only two patients included who had a perceived poor outcome, our sample size is limited in this regard.

Weaknesses of this study include it being run over two sites. Although these sites cover a population with a wide rage of sociodemographic characteristics, we found the range of ethnicities that were present within the study population limited. This likely reflects two factors. Firstly, the population the study was based in has low rates of some ethnicities [31]. Secondly, the utilization rates of orthopaedic services was lower amoung ethnic minorities, as demonstrated through the medical coding data that was used to identify some patients. Utilisation of healthcare has been found in previous studies to alter by race [38, 39]. To counteract this, the research team went to extensive lengths to ensure as much diversity as it could and managed to reflect the population demographics of the catchment areas. However, it is likely that the ethnic minority participants in the study are systematically different to those that underutilise healthcare. Compounding this is that the use of an “opt in” procedure to recruit participants inevitably leads to selection bias.

We only included patients that had osteoarthritis in the study. As this represents over 90 % of the patients who have knee replacements, and is the population of patients where the majority of work on developing an outcome prediction tool is focused, we this this was an appropriate decision.

We have compared answers from patients across different methods of data collection (focus groups and interviews). This may result in differences being apparent due to the collection method, for example people in the focus groups not feeling able to speak up, or providing a more ubiquitous point of view. Given the diversity of opinion and the personal nature of some of the information disclosed in the focus groups we do not think this was a particular issue. However, some differences may persist.

A further weakness is the power differential generated by the interviewer being an orthopaedic surgeon in training (TB). This may have led to more guarded responses. A member of the public (PS) was present for most interactions, and it was felt that this helped to allow open and honest communication [40].

This study is the first of its kind to examine outcome prediction in this way; however, a wealth of information is available on information giving strategies. Patient Decision Aids (PDA, also know as decision aids, decision support interventions, decision support aids) are methods of informing patients about the treatment options available to them [24]. From a simple one-page summary to interactive online tools, they have been widely implemented within the NHS, with the Department of Health QIPP programme funding the development and hosting of 38 PDAs to cover a variety of conditions. Knee osteoarthritis is included [40]. These aids are quite different from individualised prediction of outcome, particularly outcome based on predominantly psychological factors, and do not offer any potential for interventions based on predicted scores. However, the principle of patients weighing up probabilistic information is broadly similar.

A recent Cochrane review on the effectiveness of decision aids [41] concluded that in general they

- Improve their knowledge of the options (high-quality evidence);
- Feel more informed and more clear about what matters most to them (high-quality evidence);
- Have more accurate expectations of possible benefits and harms of their options (moderate-quality evidence); and
- Participate more in decision making (moderate-quality evidence).

Interestingly decision aids have been found to reduce the proportion of people progressing to elective surgery [24, 41]. Although these studies did not involve patients with knee osteoarthritis, there are similarities in that it is a preference-based decision [24]. There is, however, a caveat with informed decision making – patients who have knee OA tend to prefer a paternalistic interaction, commonly viewing anything else as an attempt to avoid responsibility [32–34], and shared decision making models have been shown to have mixed effects on *clinical* outcomes (rather than outcomes aimed at measuring the decision making process itself) [42].

These findings are largely consistent with our own, and are particularly relevant when considering how an outcome prediction tool should be used. It is clear that the effect of an outcome prediction tool is to a degree dependent on the content and presentation, the point in the pathway it is used, and whether it is delivering “good” or “bad” news. The work on PDAs would suggest we could expect a decrease in the proportion of people progressing to elective surgery. Additionally how the tool is delivered is likely to be key (i.e. as part of a shared decision making model or part of a paternalistic model). These uncertainties will need careful evaluation if and when such a tool becomes available.

## Conclusion

This study has demonstrated that the timing of delivery of predictive information, along with the optimism bias, will have a large effect on any future tool capable of predicting outcome. The implications from this, in the authors’ opinion, is that the timing and effect (both in terms of decision making and clinical outcome) will have to be carefully evaluated for any potential outcome prediction tool that is to be used by patients.

## Additional files

Additional file 1: Outcome prediction report. An example of a fictitious outcome prediction report. (DOCX 1259 kb) Additional file 2: Focus group guide. (DOCX 25 kb) Additional file 3: Interview group guide. (DOCX 23 kb)
