# Supplementary material for: How outcome prediction could affect patient decision making in knee replacements: a qualitative study
Source: BMC Musculoskelet Disord. 2016 Jul 22;17:304. doi: 10.1186/s12891-016-1165-x (PMC4957427; doi:10.1186/s12891-016-1165-x)
Supplement: Additional file 1: — Outcome prediction report. An example of a fictitious outcome prediction report. (DOCX 1259 kb) [file 12891_2016_1165_MOESM1_ESM.docx]

Example of an outcome prediction report

On the following pages there is an example of an outcome prediction report for a fictitious patient – Joe Bloggs.

To get to the stage of reading this report, Joe Bloggs will have completed an extensive questionnaire pack. The information from the questionnaires will be used to generate the report.

This report should give you an idea of the kind of information we could include on an outcome prediction report. We are trailing different ways of presenting the information, and would be very interested in any ideas you have that would make the presentation clearer.

We would also be interested in any aspects of this report that do not make sense to you.

A full description of what an outcome prediction tool is, and how it works, will be provided on the day.

The Knee Outcome Report

Prepared for: Joe Bloggs

Date: 9/12/2013

# Contents

The knee outcome report – What does it mean . . . . . . . . . . . . . 3

Knee outcome report – the bottom line . . . . . . . . . . . . . . . . . . . 5

Knee outcome – Pain . . . . . . . . . . . . . . . . . . . . . . . . . . . . . . . . . 6

Knee outcome – Function . . . . . . . . . . . . . . . . . . . . . . . . . . . . . .8

The knee outcome report – what does it mean?

This report is designed to help you come to a decision regarding knee replacement surgery. If there is anything that you do not fully understand don’t be afraid to ask.

You can discuss the outcome of this report with a health professional, who will be able to guide you through the information.

# What is the aim of the report?

- To give an estimate of what you, as an individual, can expect from a knee replacement

# What is in the report?

This report gives you an **estimate** on what you can expect from a knee replacement:

- It gives you the “bottom line” of what you can expect
- It gives a more detailed estimate on whether you will have any long term pain
- It gives a more detailed estimate of how well your knee will work (knee function) after a knee replacement

**Different people will want different things from a knee replacement – for example one persons priority may be pain relief, while another may want to be able to walk to the shops.**

**Therefore, it is up to you to decide if the improvement that we have predicted is worthwhile for you.**

# How do you generate the report?

The report is generated through a detailed questionnaire that you have completed.

# What do I do now?

We recommend that you discuss the report, and the pros and cons of an operation, with a healthcare provider. You will then be in a position to make an informed decision about a knee replacement.

The bottom line

# One year after the operation:

## There is a 30% chance you would not have had the operation if you could go back in time.

This means that if 100 people in your position had a knee replacement, 30 would not have the operation if they could go back in time

## There is a 20% chance you would not recommend a knee replacement to a friend of member of your family.

This means that if 100 people in your position had a knee replacement, 20 would not recommend the treatment to a friend or member of their family.

## There is a 15% chance you will still have pain in your knee after the operation.

This means that if 100 people in your position had a knee replacement, 15 would still have pain after the operation.

## Your knee will work better after a knee replacement – this improvement is likely to be below average.

This means that most people in your position would improve more that you would. However, a small improvement may be very worthwhile to some patients, whereas a large improvement may not be worthwhile to another. It is up to you as an individual (with help from your surgeon) to decide if the operation is worthwhile.

Knee outcome report – Pain

# Knee pain

Some patients have pain in their knee after they have had a knee replacement.

**It is important to note that some patients may have a low chance of having pain and still develop it, and some patients may have a high chance and not develop pain.**

### Your chance of developing mild pain is 10%

This means that if 100 people in your category received a knee replacement, 10 would develop mild pain.

### Your chance of developing moderate to severe pain is 5%

This means that is 100 people in your category received a knee replacement, 5 would develop moderate to severe pain.

These chances are demonstrated in the diagram below

**
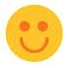
**

Patient without significant pain after knee replacement

Patient with mild pain after knee replacement


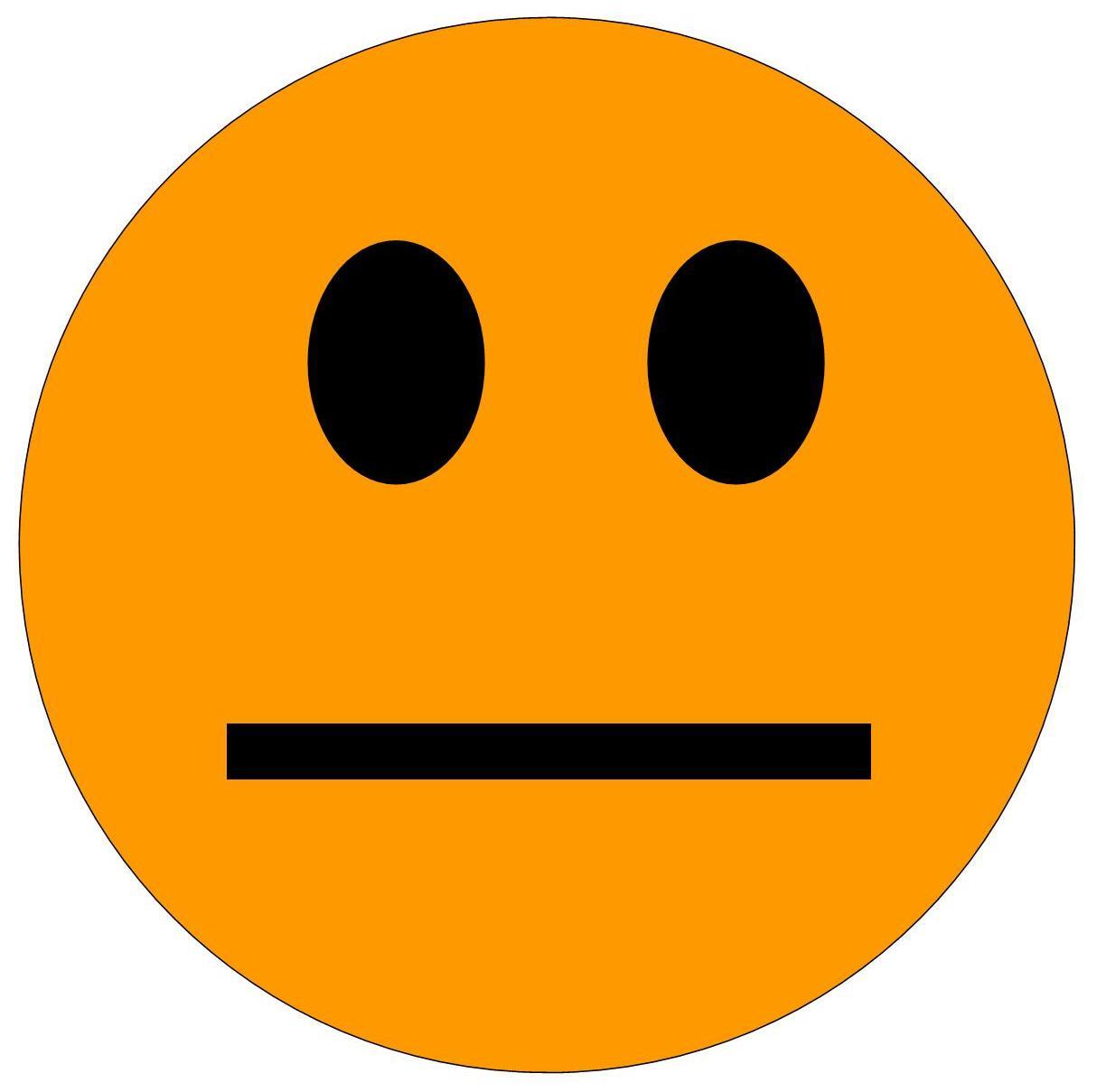


**
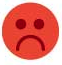
**

| **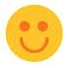** | **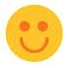** | **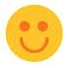** | **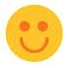** | **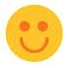** | **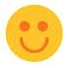** | **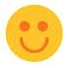** | **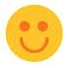** | **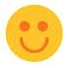** | **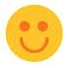** |
| --- | --- | --- | --- | --- | --- | --- | --- | --- | --- |
| **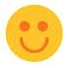** | **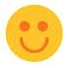** | **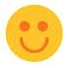** | **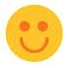** | **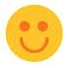** | **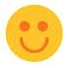** | **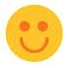** | **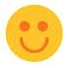** | **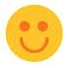** | **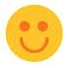** |
| **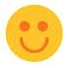** | **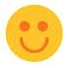** | **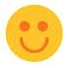** | **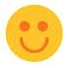** | **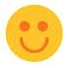** | **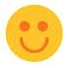** | **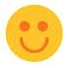** | **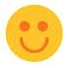** | **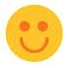** | **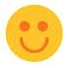** |
| **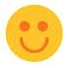** | **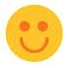** | **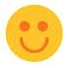** | **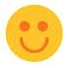** | **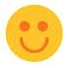** | **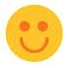** | **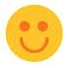** | **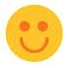** | **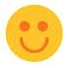** | **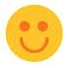** |
| **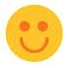** | **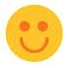** | **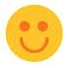** | **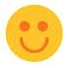** | **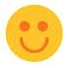** | **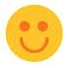** | **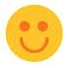** | **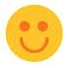** | **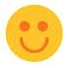** | **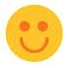** |
| **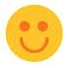** | **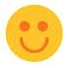** | **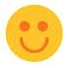** | **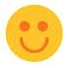** | **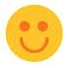** | **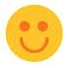** | **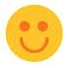** | **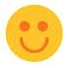** | **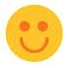** | **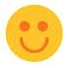** |
| **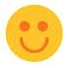** | **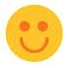** | **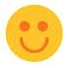** | **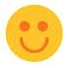** | **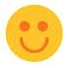** | **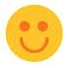** | **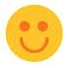** | **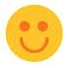** | **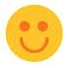** | **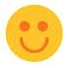** |
| **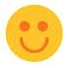** | **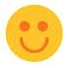** | **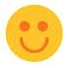** | **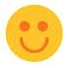** | **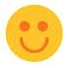** | **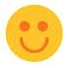** | **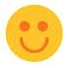** | **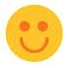** | **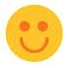** | **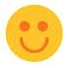** |
| 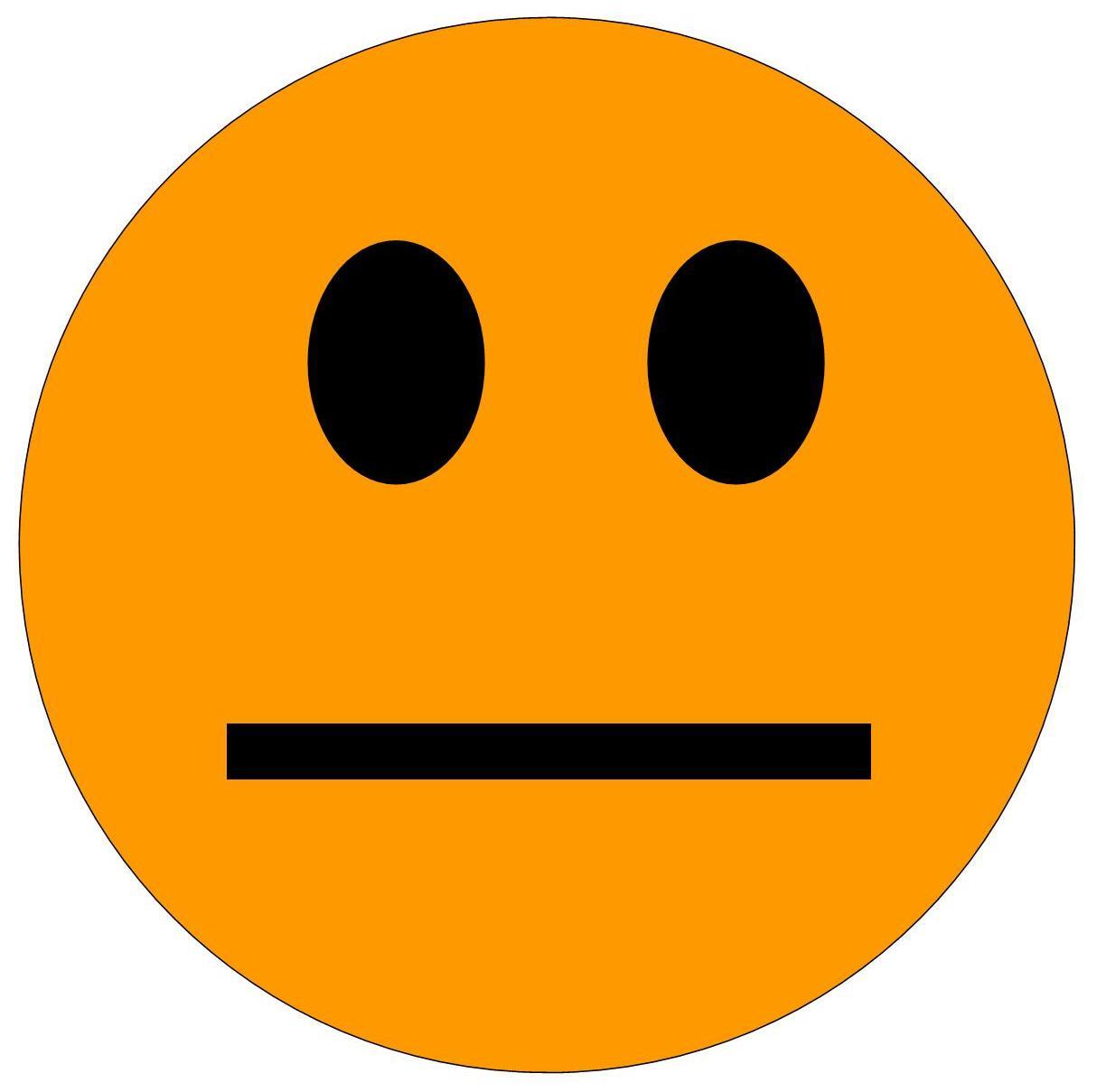 | 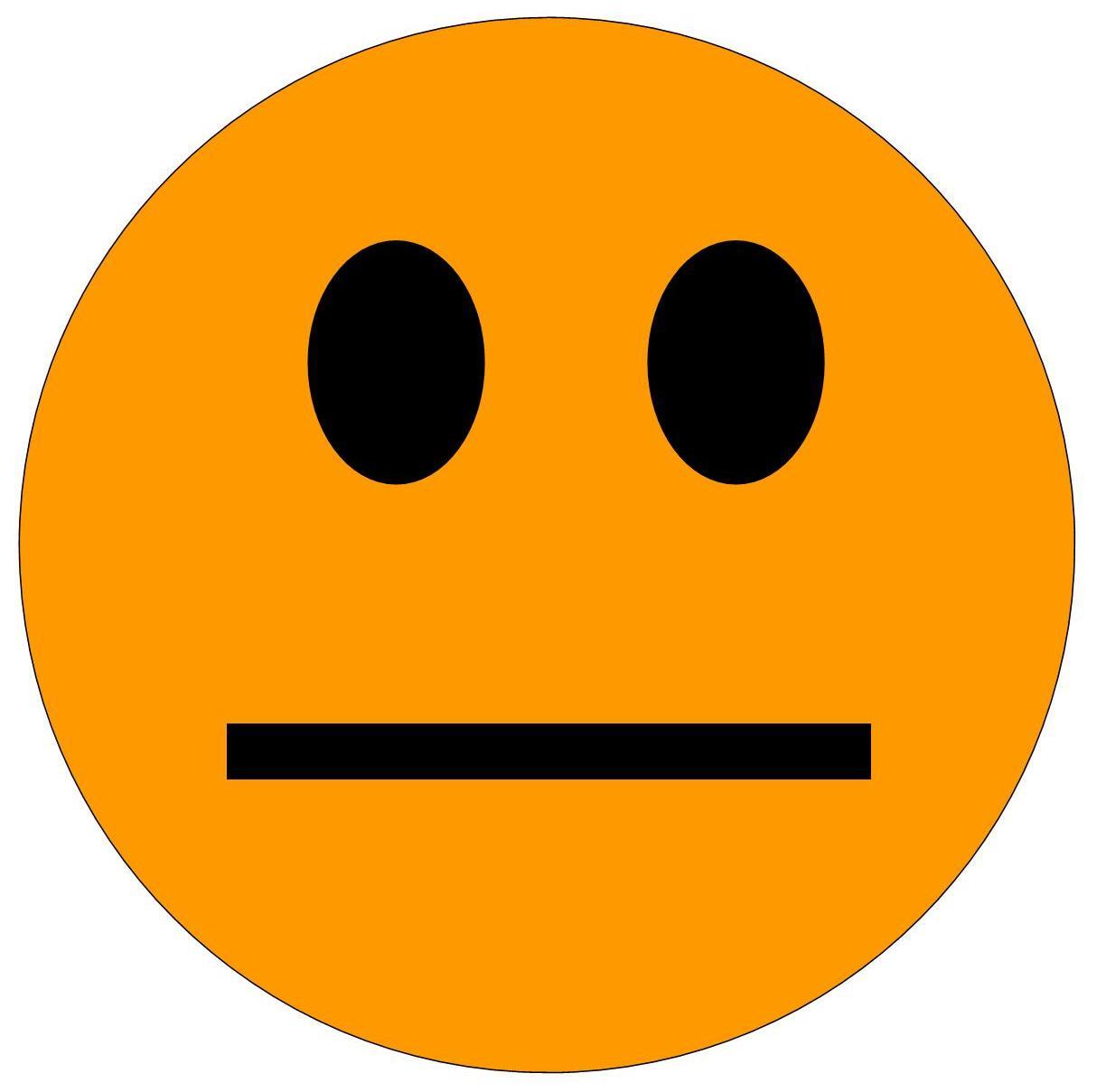 | 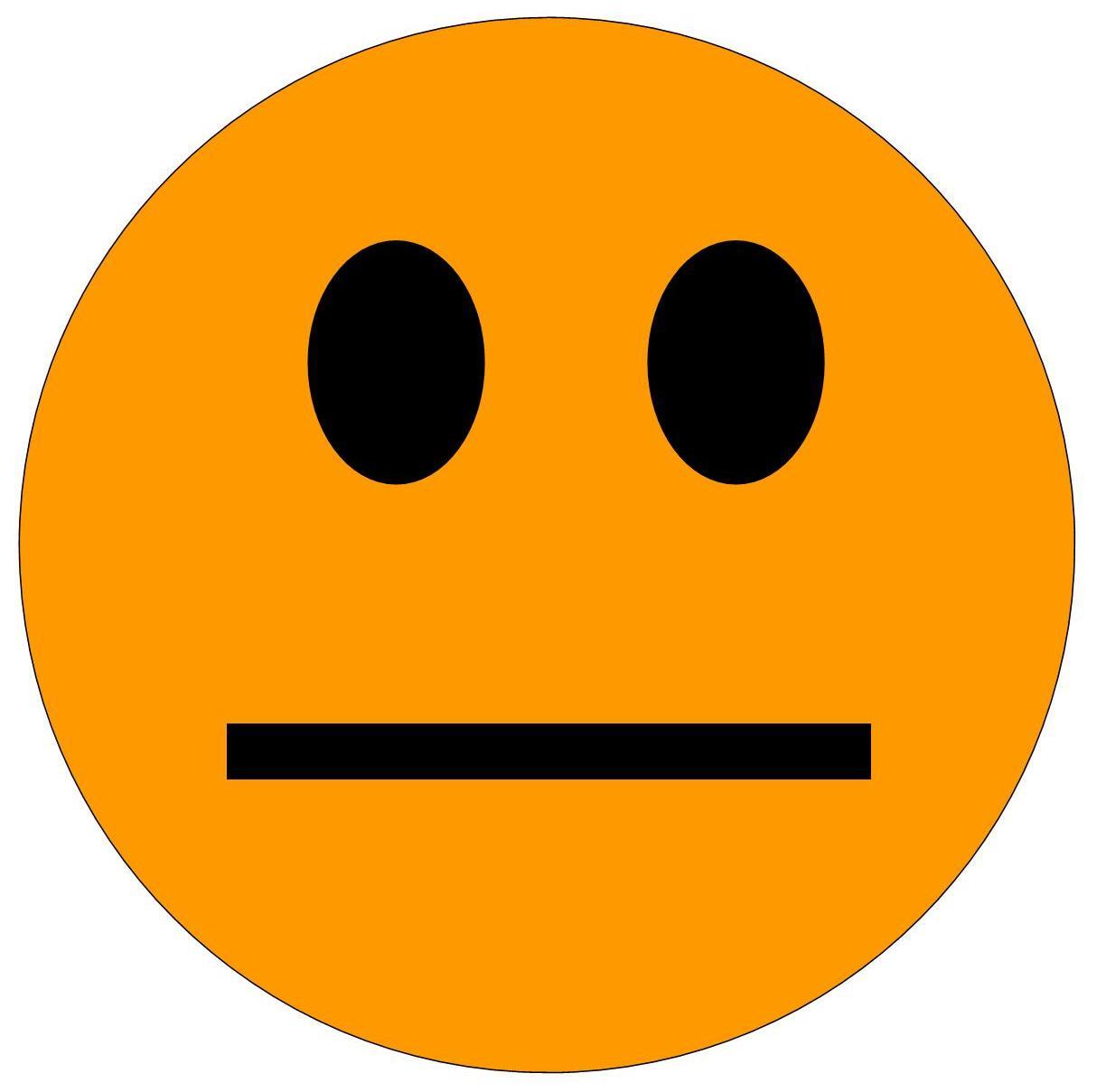 | 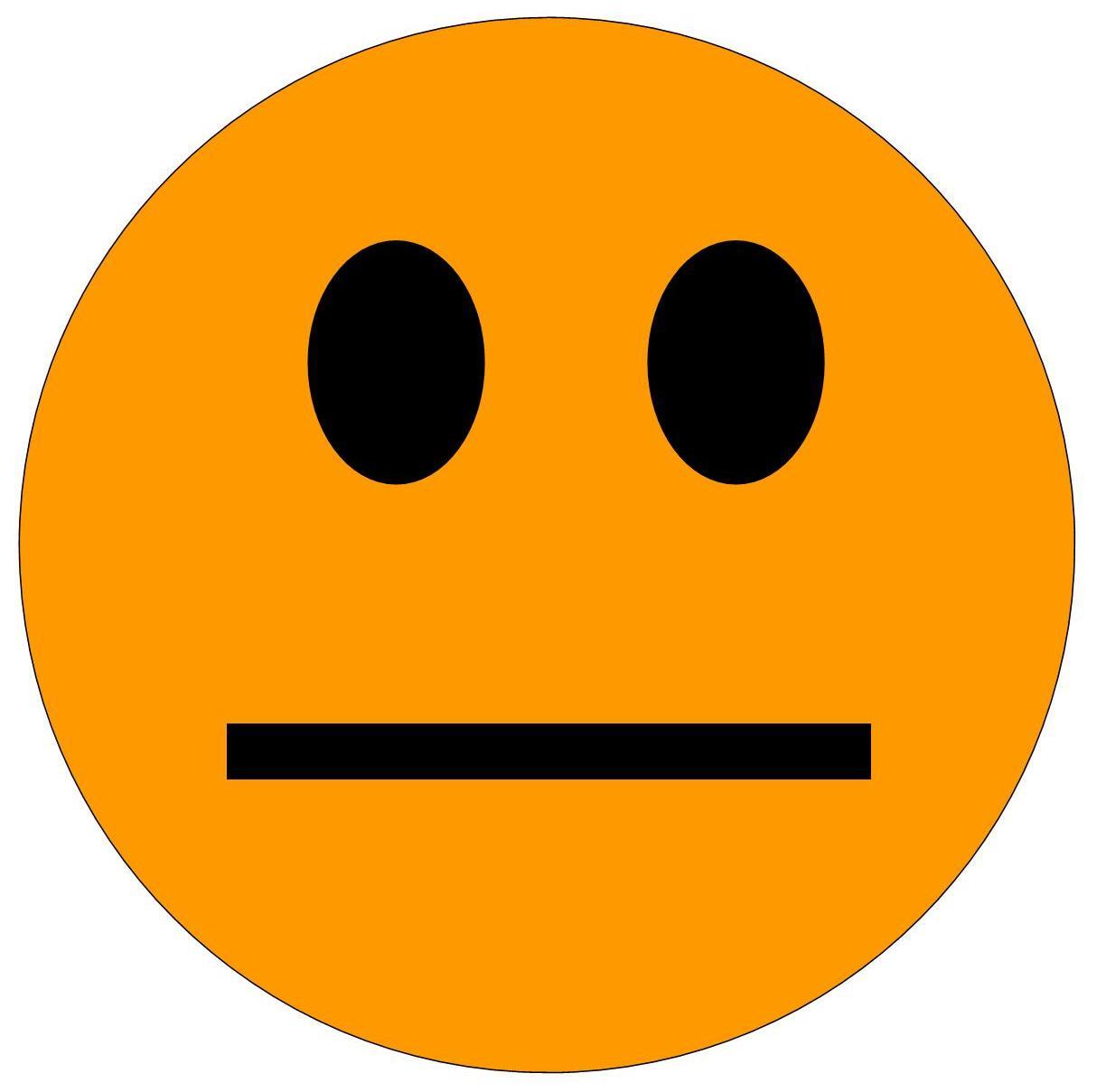 | 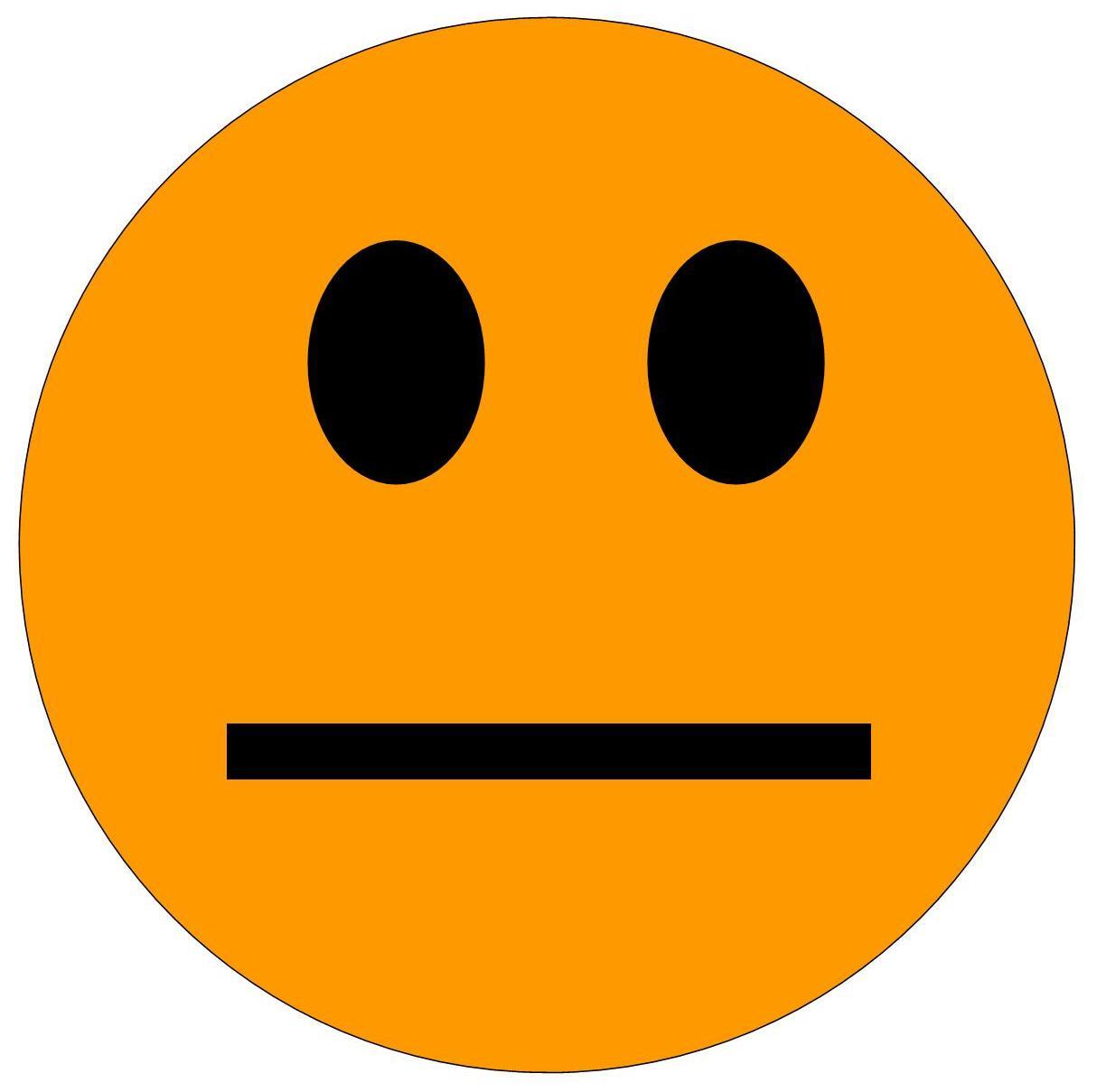 | 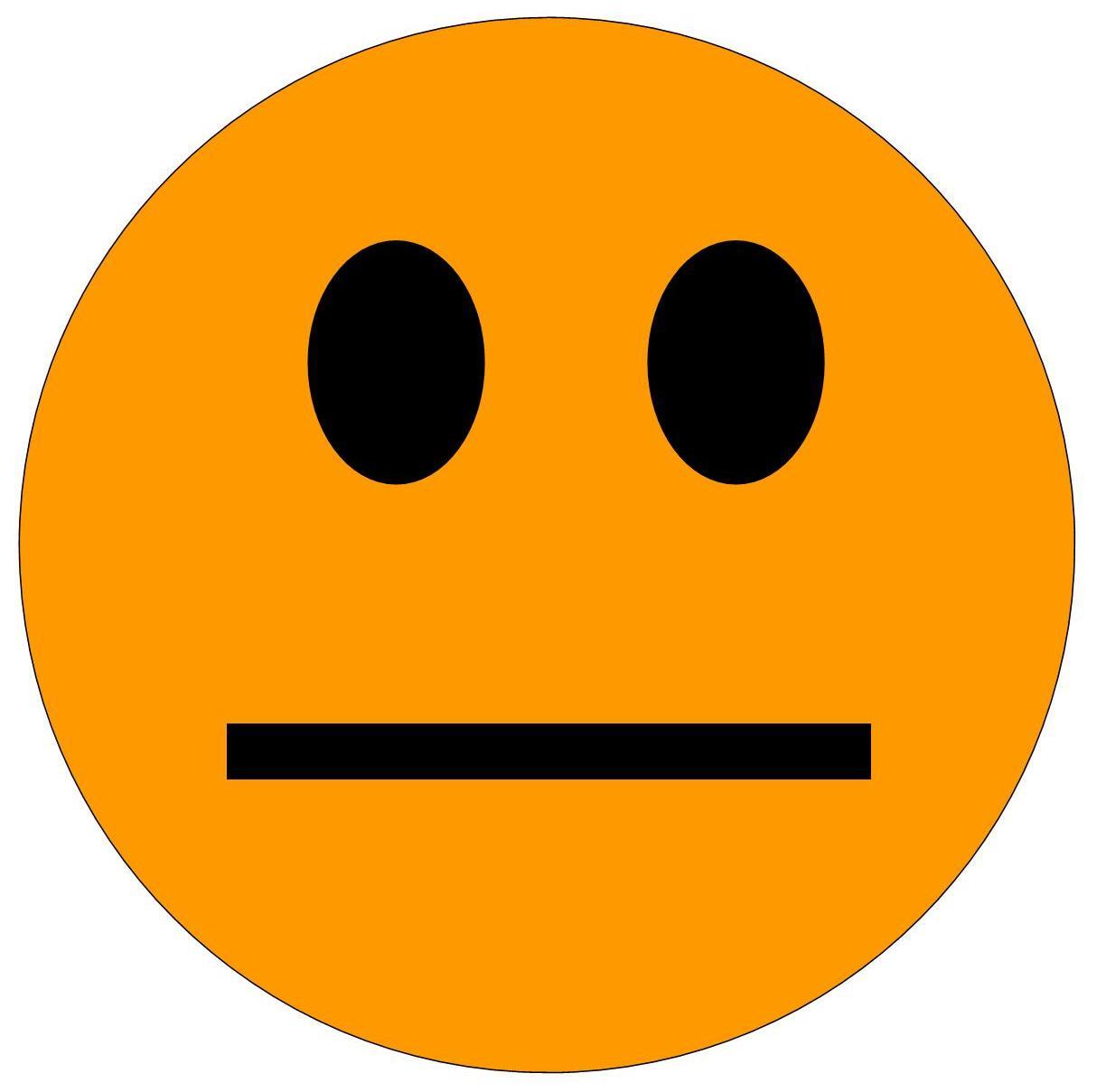 | 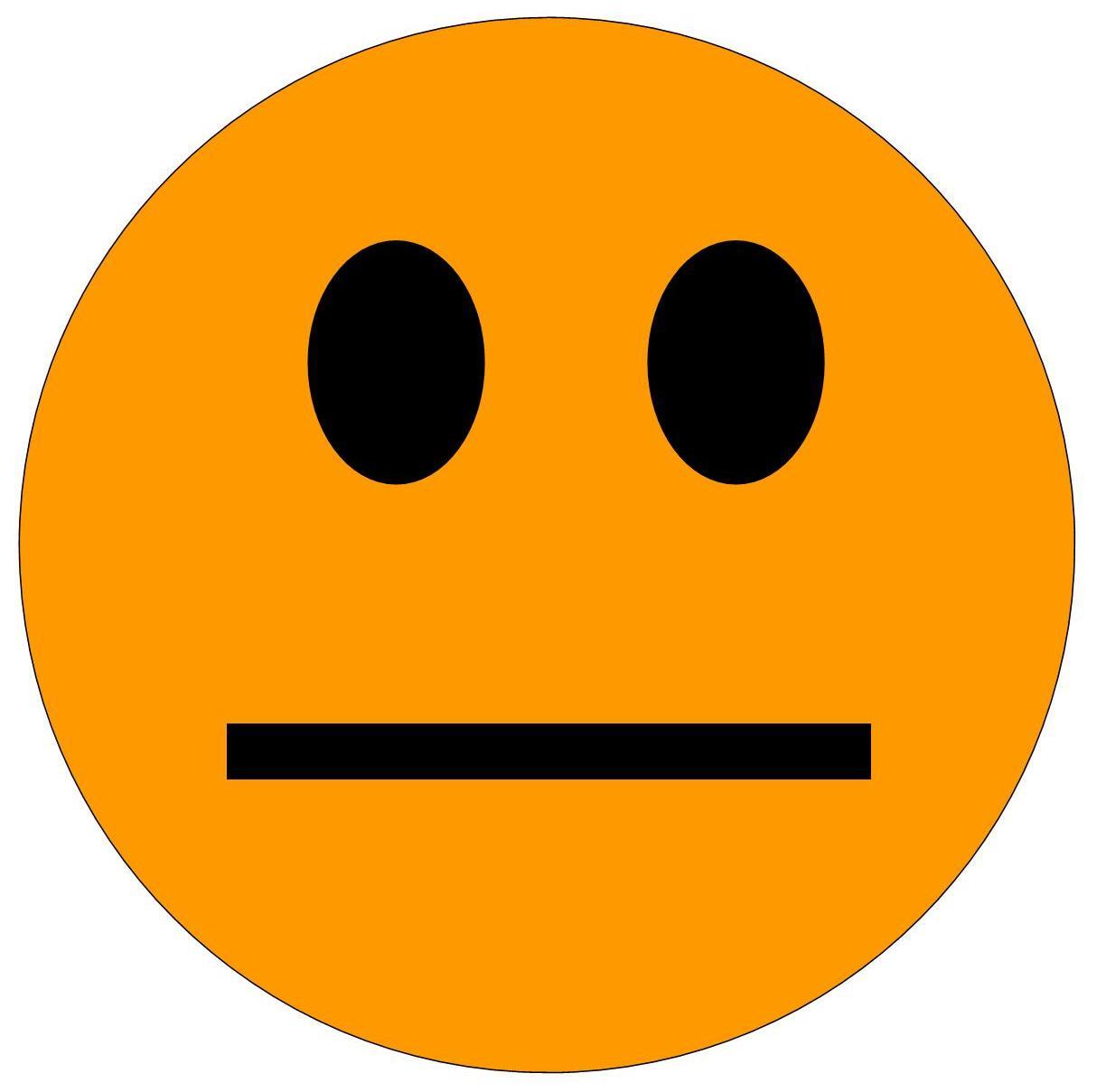 | 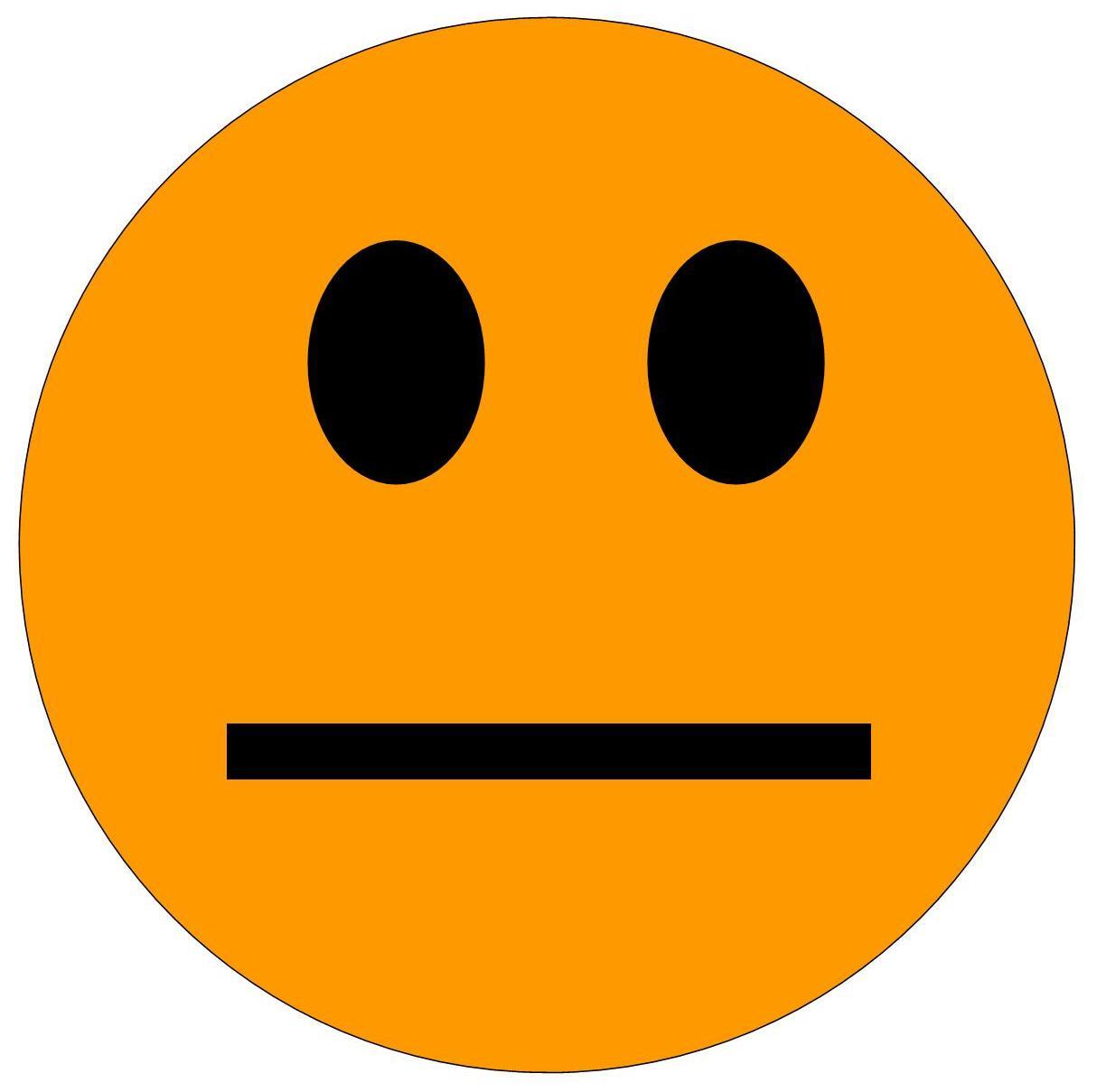 | 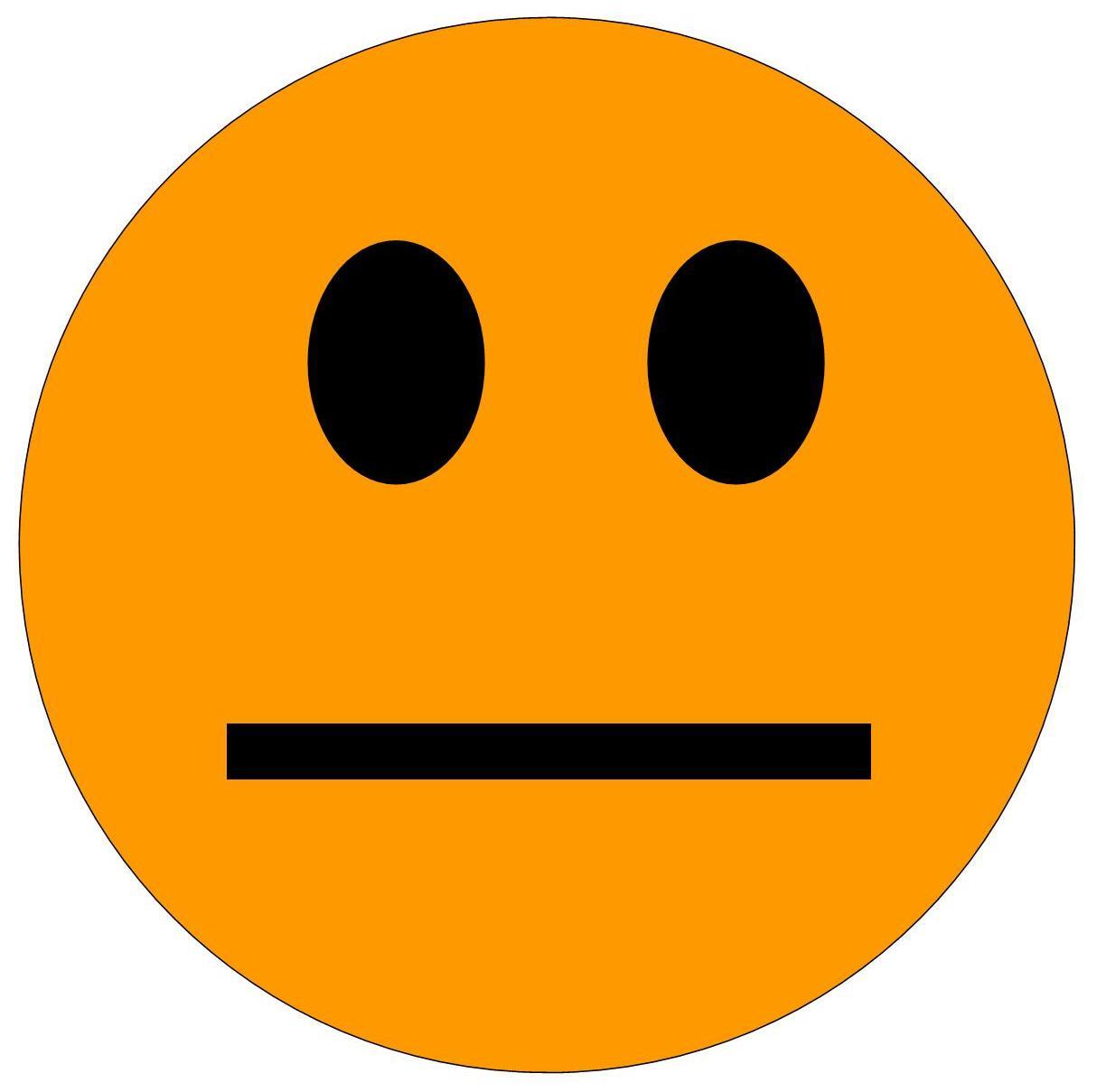 | 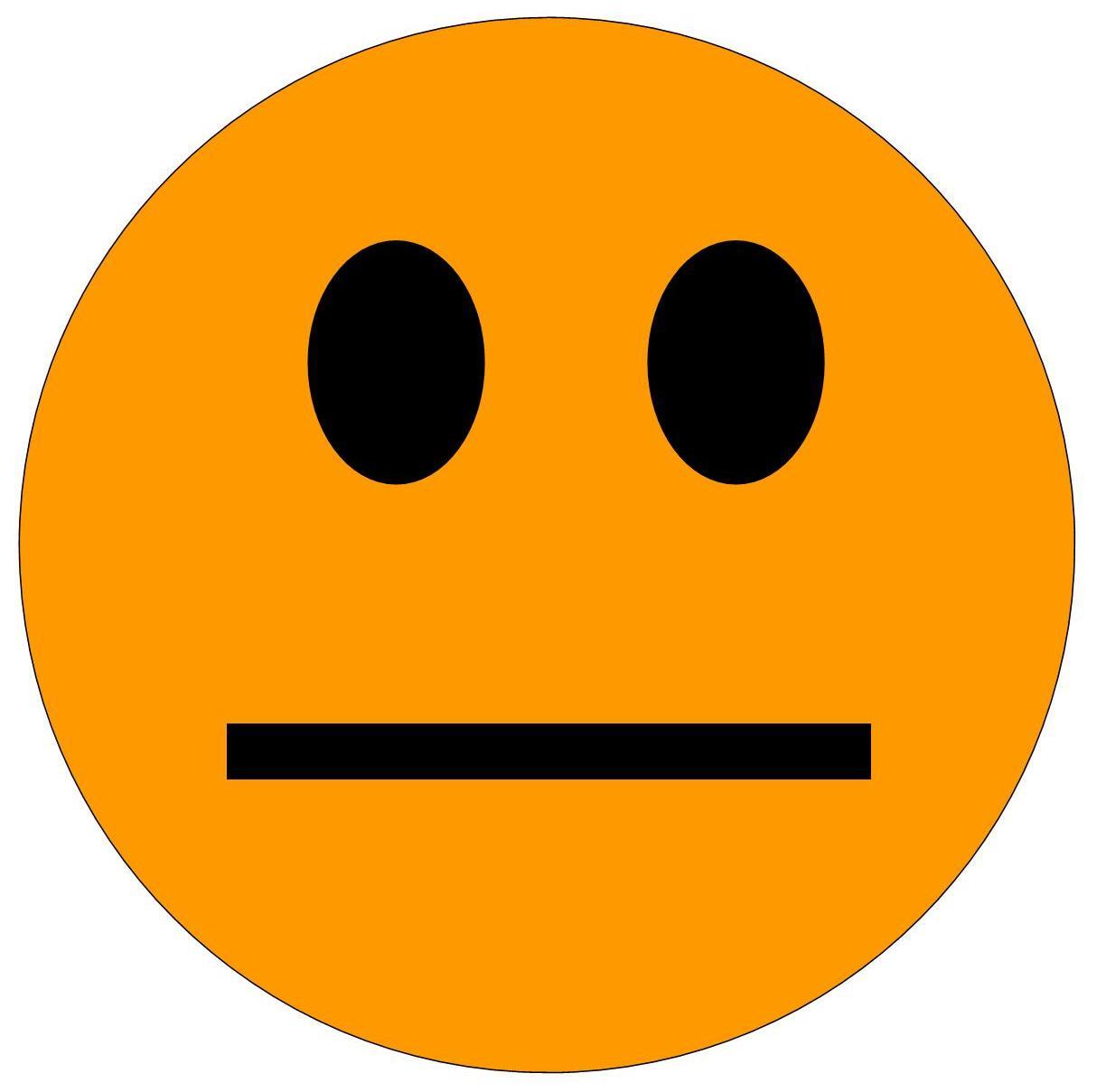 |
| 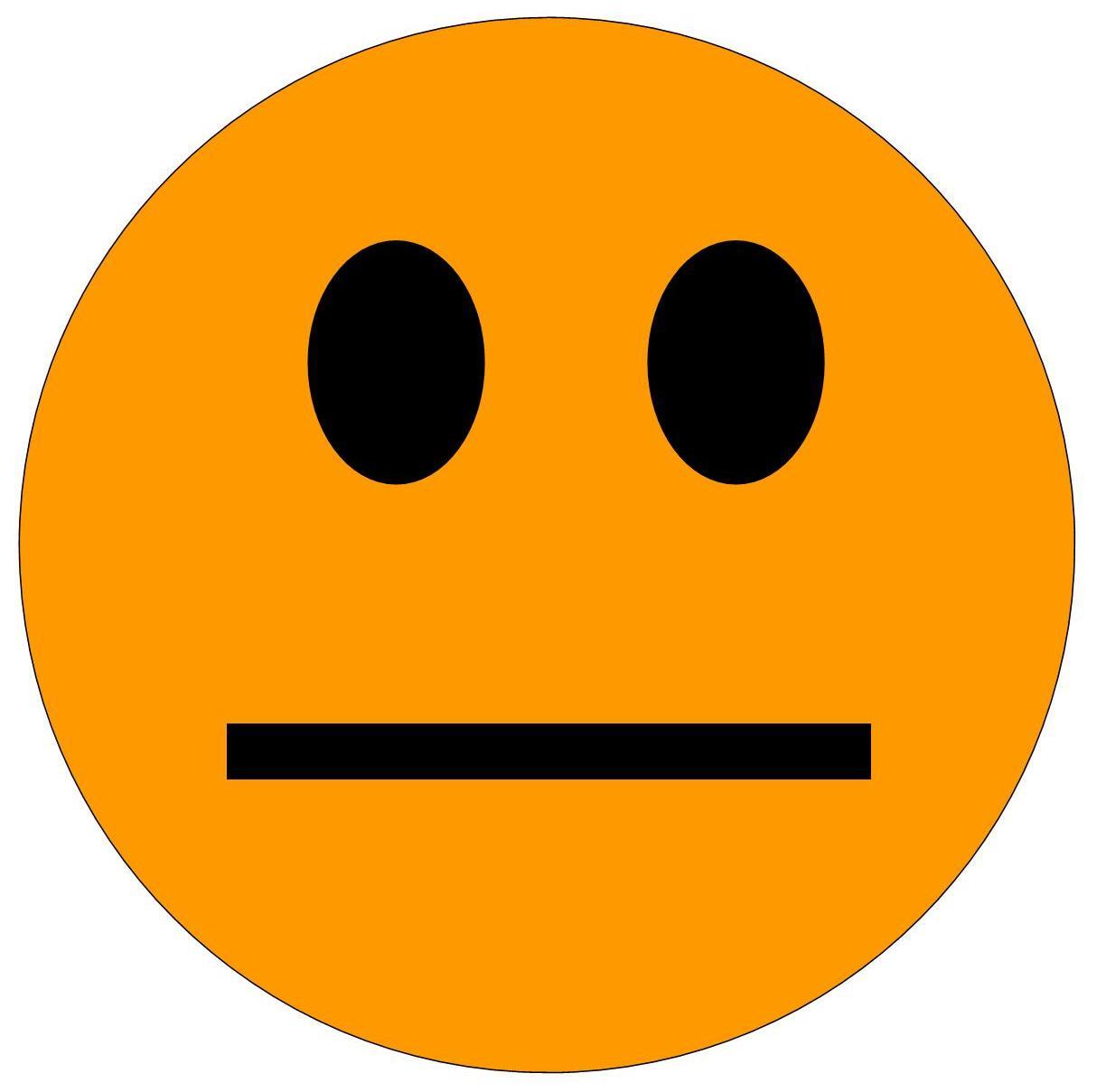 | 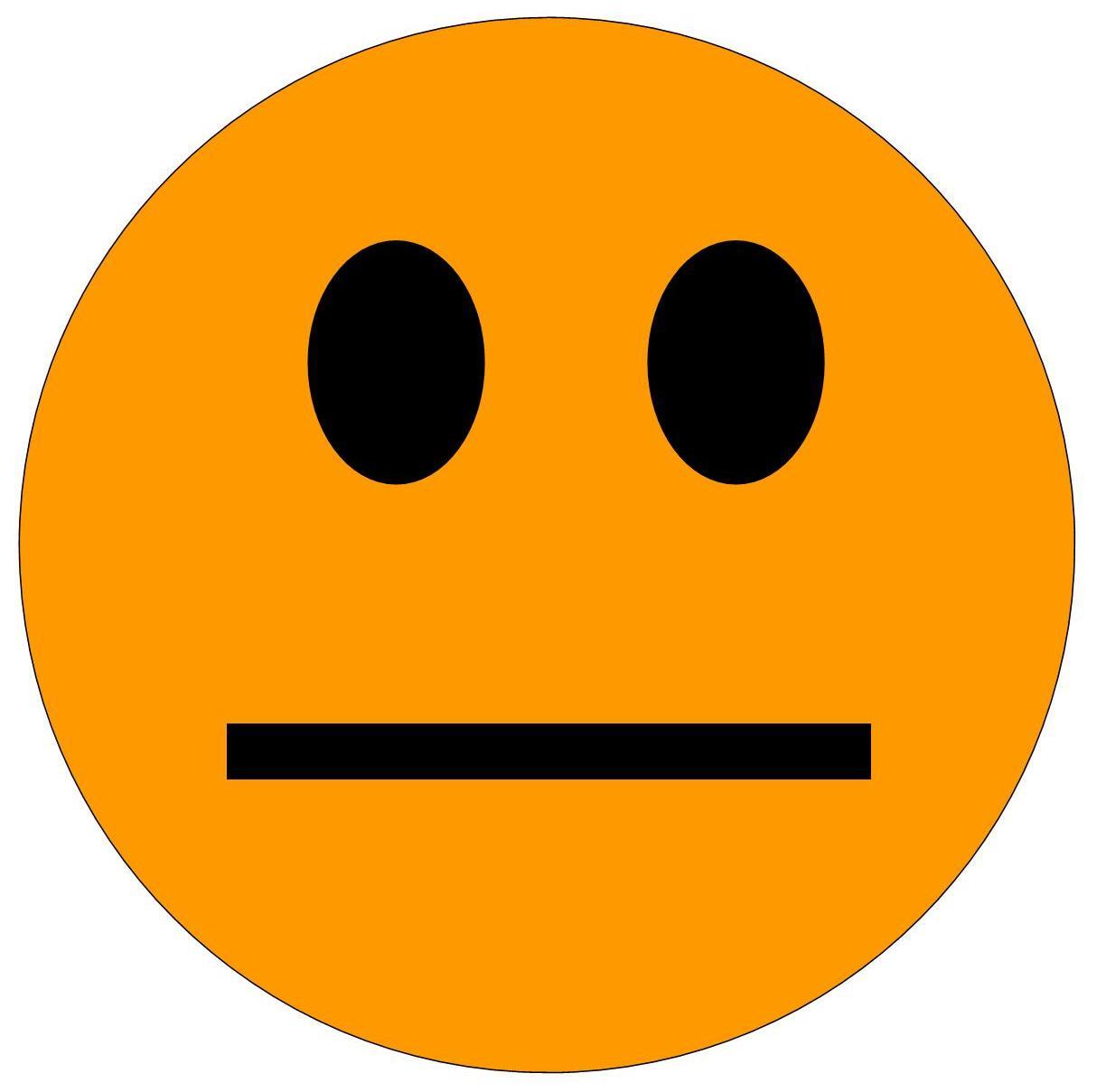 | 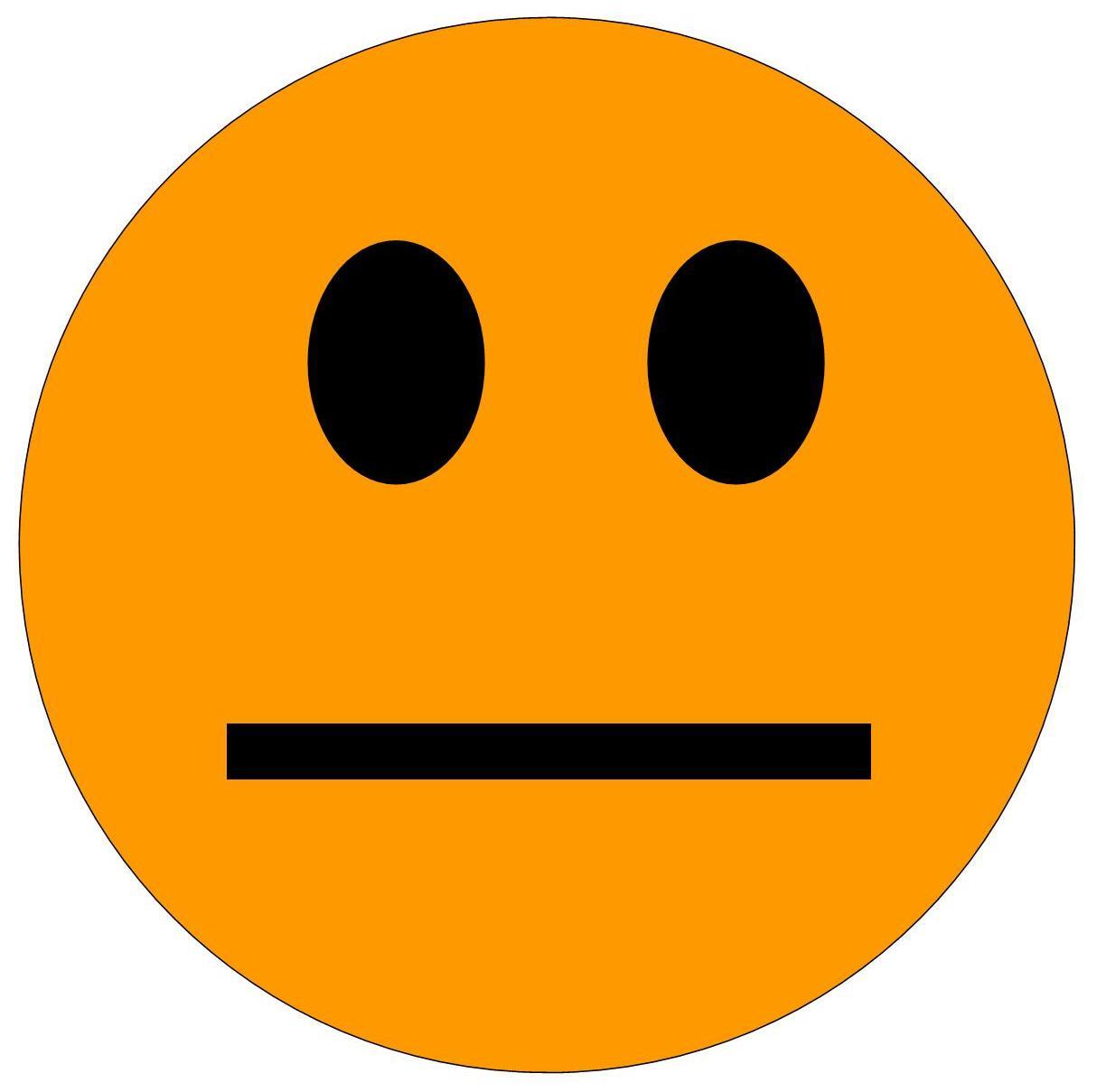 | 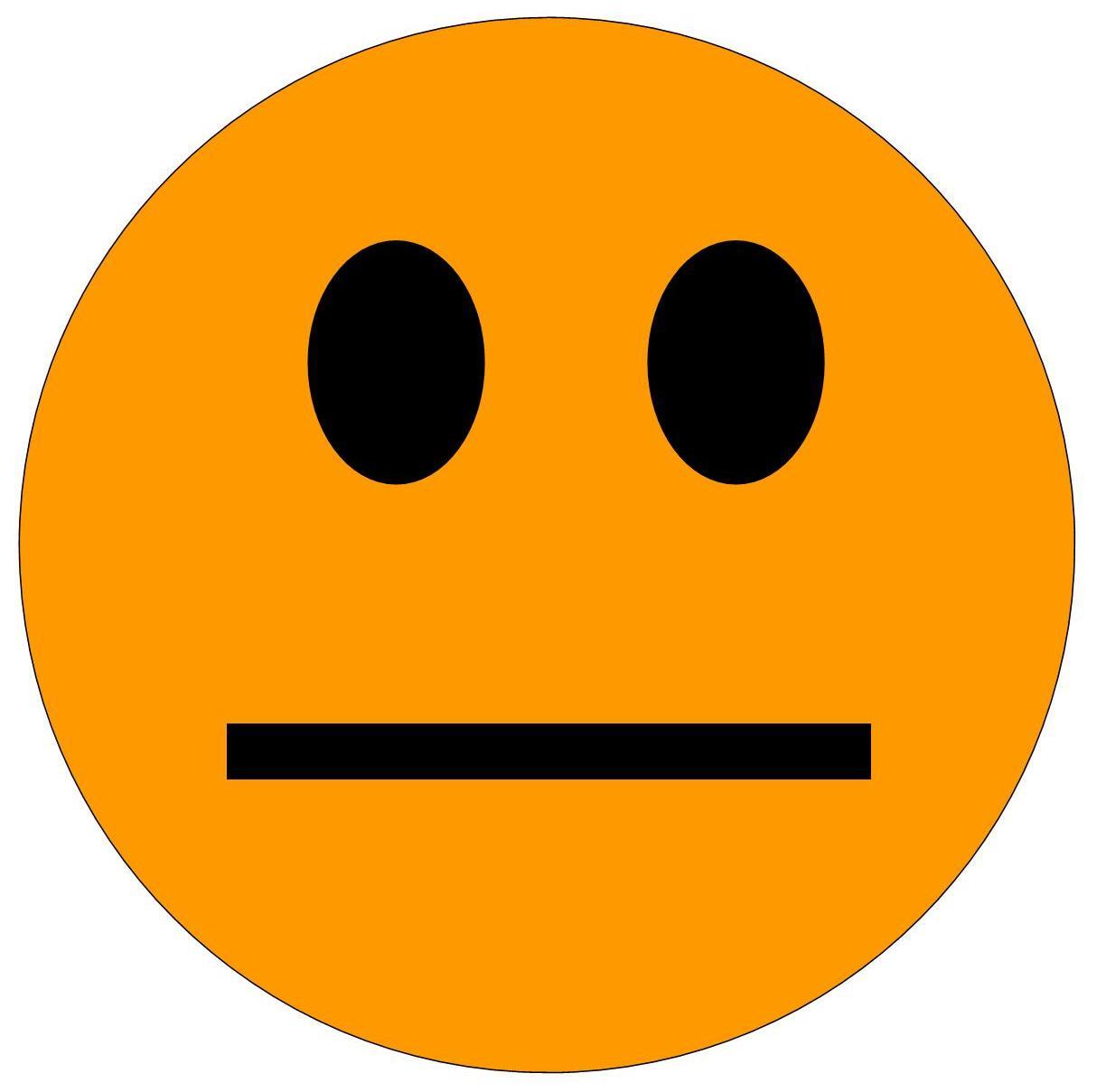 | 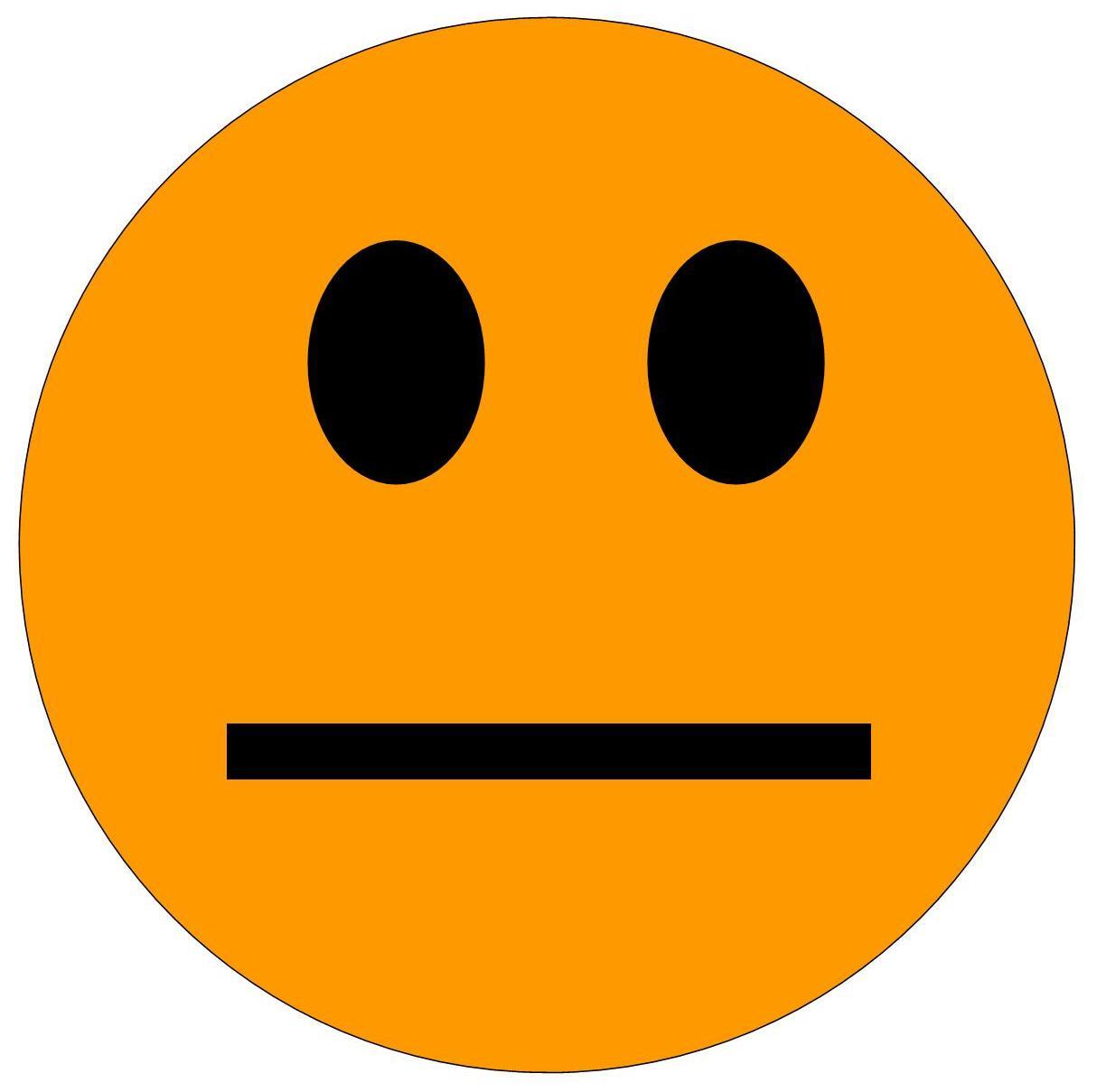 | **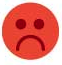** | **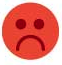** |  |  |  |

Patient with moderate to severe pain after knee replacement

Knee outcome report – Function

How your knee works (e.g. climbing stairs) ***before*** a knee replacement varies from person to person. Therefore, to get an idea of how much your knee may improve, you need to be able to know what it is like before an operation.

We have assed how your knee is works ***currently*** (before your knee replacement) using a scoring system. This is reported on a scale running from 1 (very poor function), to 100 (very good function). Your score is:

50

We expect your score (and therefore how well your knee works) to improve after a knee replacement. Some patients improve more than others.

Some patients find a small improvement in their score results in a **significant improvement in their quality of life. It is difficult to predict exactly how a knee replacement will affect you personally.**

We expect your score to improve by:

10 POINTS

What this means to you is explained in the following pages.

We expect your knee function to improve by about 10 points. This is below average for a knee replacement.

**A small improvement in knee function may be very worthwhile for one patient, but not for another. A small improvement on the scale can result in a large improvement in quality of life.**

**It is important to note that an improvement from 40 to 50 is not the same as an improvement from 50 to 60 – what is important is where you are starting from, and where you end up. This is explained in the following pages.**

Your score before a knee replacement

Your score after a knee replacement

9 0

8 0

7 0

6 0

5 0

4 0

3 0

2 0

1 0

100

**Worst**

**Possible knee function**

0

**Best**

**Possible knee function**

# What does this mean for me?

To help understand what an improvement of 10 points could mean for you we have provided an example below.

**These are examples only. We are not able to predict each individual item, and you are likely to score higher, or lower, for several of them.** However, the **overall** improvement is likely to be similar.

Each item below shows how you answer the question now, and how you are likely to answer it one year after the operation.

## During the past 4 weeks, how would you describe the pain you usually have from your knee?

## During the past 4 weeks, have you had any trouble with washing and drying yourself (all over) because of your knee?

## During the past 4 weeks, have you had any trouble getting in and out of a car or using public transport because of your knee?

## During the past 4 weeks, for how long have you been able to walk before pain from your knee becomes severe?

## During the past 4 weeks, after a meal (sat at a table), how painful has it been for you to stand up from a chair because of your knee?

## During the past 4 weeks, have you been limping when walking because of your knee?

## During the past 4 weeks, could you kneel down and get up again afterwards?

## During the past 4 weeks, have you been troubled by pain from your knee in bed at night?

## During the past 4 weeks, how much has pain from your knee interfered with your usual work (including housework)?

## During the past 4 weeks, have you felt that your knee might suddenly “give way” or let you down?

## During the past 4 weeks, could you do the household shopping on your own?

## During the past 4 weeks, could you walk down one flight of stairs?

# Should you have any questions or concerns regarding this report please discuss it with one of your healthcare providers.
