# Supplementary material for: How outcome prediction could affect patient decision making in knee replacements: a qualitative study
Source: BMC Musculoskelet Disord. 2016 Jul 22;17:304. doi: 10.1186/s12891-016-1165-x (PMC4957427; doi:10.1186/s12891-016-1165-x)
Supplement: Additional file 3: — Interview group guide. (DOCX 23 kb) [file 12891_2016_1165_MOESM3_ESM.docx]

Interviews – Outcome prediction tool

# WELCOME

Thanks for agreeing to take part. We appreciate your willingness to participate.

INTRODUCTIONS

PURPOSE OF INTERVIEW

This interview is aimed at understanding how a new tool, which we will explain fully later, might have influenced your decisions.

We would like to record the conversation. This recording will be used by the research team to ensure we don’t miss anything. It will be transcribed (typed out) for us to analyse. We will ensure it is kept confidential at all times, and we will destroy the recording once the transcripts have been prepared. You will have the opportunity to look through the transcript and let us know if you would like to change anything.

Some things you say may be used in a publication with your permission; however, we will make every effort to ensure you remain anonymous.

I expect this interview to take around 20-30 minutes. If, at any time, you feel tiered or want to stop please tell us.

## Personal situation and physical state

Can you tell us a little about yourself, and the events that lead to you being listed for a knee replacement please?

# Introduce OPT -- What is an outcome prediction tool

The outcome after knee replacement is variable – it is safe to say that almost all patients improve after a knee replacement; however, some patients improve more than others. We measure this improvement in various ways, but two of the most common are: function (i.e. how well the knee does things we want it to, like climbing stairs and getting into and out of a car – this is normally measured via questionnaires); the second is pain – some people have ongoing pain after a knee replacement.

Currently, we are not able to tell which patients will do very well from a functional point of view (up and down stairs, kneeling), and which patients will have ongoing pain. Patients who have ongoing pain tend to have worse function.

We are currently developing a questionnaire that will be able to predict outcome – i.e. it will be able to tell which patients are likely to develop pain and what functional improvement a patient can expect. This is what we mean by outcome prediction tool.

Do you have any questions?

## What do you think of the outcome prediction tool?

I would like to start by going through a couple of examples of how an outcome prediction tool could look. I hope this will help you understand the type of information that will be included, and we would be interested in your views on how it is presented, and how easy it is to understand.

Prompts

- Do you find the charts helpful?
- Do you find identifying which patient you are helpful?
- Are rating things into categories a good idea?
- Do you understand the information – is there anything that could be improved?
- Is there too much information or too little?
- Is there too much detail or too little?

## Do you think that having a prediction of the outcome would have been helpful to you?

Prompts

- Would you have trusted it?
- Would it have altered your decision (either way)?
- Would it have altered your expectations?
- Would it have affected your confidence or fear?
- Would it have given you more confidence to go ahead?
- If the outcome was good would you want an earlier TKR?
- IF you had this information, would you have not gone to, for example, your family or friends, or a website. Why?

## Does an OPT alter the fear of the procedure itself?

## Who do you think should decide if you have a knee replacement?

Prompts:

- Should the surgeons decide?
- Should you have access to it whenever you like?
- Do you think that things going on just now limit your ability to decide (e.g. struggling to do certain things with the knee (cope), fear of the operation, pain).

## How would you have coped if you had a poor outcome prediction? (Est time 10 min)

Prompts

- Suspicious?
- Not believe?
- Take it personally – would it feel like a personal attack?
- Would it feel like a diagnosis?
- Do you think it would help you cope after the operation?

Need to push here on the use of psychological factors. May need an explanation at this point, and an exploration of how they answer the above questions in the light of that.

## Possibility of delaying Knee Replacment to modify other factors (e.g. CBT for coping strategys). What are your views on this?

Prompts

- Acceptable
- What if outcome is still poor
- Would you have been amenable to CBT or Motivational interviews or physio/increased physical exercise.

## Who do you think should do the outcome prediction tool – the patient himself or herself, with the GP, a specialist nurse, or when they see the surgeon?

## Is there anything else you would like to say about outcome prediction in knee replacements?
